# The development, implementation and evaluation of interventions to reduce workplace sitting: a qualitative systematic review and evidence-based operational framework

**DOI:** 10.1186/s12889-018-5768-z

**Published:** 2018-07-04

**Authors:** Kelly Mackenzie, Elizabeth Such, Paul Norman, Elizabeth Goyder

**Affiliations:** 1 School of Health and Related Research, University of Sheffield, Regent Street, Sheffield, S1 4DA UK; 2 Department of Psychology, University of Sheffield, Cathedral Court, 1 Vicar Lane, Sheffield, S1 2LT UK

**Keywords:** Sedentary behaviour, Sitting time, Workplace, Occupation, Intervention development, Implementation, Evaluation, Framework, Qualitative systematic review

## Abstract

**Background:**

Prolonged sitting is associated with increased risks of cardiovascular disease, Type 2 diabetes, some cancers, musculoskeletal disorders and premature mortality. Workplaces contribute to a large proportion of daily sitting time, particularly among office-based workers. Interventions to reduce workplace sitting therefore represent important public health initiatives. Previous systematic reviews suggest such interventions can be effective but have reported wide variations. Further, there is uncertainty as to whether effectiveness in controlled trials can be replicated when implemented outside the research setting. The aims of this review are to identify factors important for the implementation of workplace sitting interventions and to translate these findings into a useful operational framework to support the future implementation of such interventions.

**Methods:**

A qualitative systematic review was conducted. Four health and social science databases were searched for studies set in the workplace, with office-based employees and with the primary aim of reducing workplace sitting. Extracted data were primarily from author descriptions of interventions and their implementation. Inductive thematic analysis and synthesis was undertaken.

**Results:**

Forty studies met the inclusion criteria. Nine descriptive themes were identified from which emerged three higher-order analytical themes, which related to the *development*, *implementation* and *evaluation* of workplace sitting interventions. Key findings included: the importance of grounding interventions in theory; utilising participative approaches during intervention development and implementation; and conducting comprehensive process and outcome evaluations. There was a general under-reporting of information relating to the context within which workplace sitting interventions were implemented, such as details of local organisation processes and structures, as well as the wider political and economic landscape, which if present would aid the translation of knowledge into “real-world” settings.

**Conclusions:**

These findings provided the basis for an operational framework, which is a representation of all nine descriptive themes and three higher-order analytical themes, to support workplace sitting intervention development, implementation and evaluation. Once tested and refined, this framework has the potential to be incorporated into a practical toolkit, which could be used by a range of organisations to develop, implement and evaluate their own interventions to reduce workplace sitting time amongst staff.

**Electronic supplementary material:**

The online version of this article (10.1186/s12889-018-5768-z) contains supplementary material, which is available to authorized users.

## Background

Sedentary behaviour, defined as any waking behaviour with an energy expenditure of ≤1.5 Metabolic Equivalents while in a sitting, reclining or lying posture [1], when carried out for prolonged periods of time has been identified as an important public health concern [2]. Prolonged sedentary behaviour has been shown to be associated with an increased risk of a range of health issues, including: cardiovascular disease [3–5]; metabolic syndrome/type 2 diabetes [3, 5–8]; obesity [9]; hypertension [10]; some cancers [11]; depression [12]; musculoskeletal problems [13]; and premature mortality [5, 8, 14]. Sedentary behaviour is most commonly assessed as time spent sitting.

Over recent decades, particularly in the Global North, the workplace has been increasingly associated with prolonged sitting behaviours due to advances in technology and computer-based tasks [15]. Observational studies suggest that office-based workers in England spend more than 60% of their total daily sitting time at work [16]. In response, a number of interventions to reduce workplace sitting have been developed and tested. Systematic reviews investigating the effectiveness of workplace sitting interventions have produced mixed results [17–22]. These reviews suggest that, at least in the short-term, intervention strategies that use sit-stand/standing desks, either as alone or as part of a multi-component intervention, may be effective, although the reductions in workplace sitting time are variable [17–22]. Ergonomic interventions such as sit-stand desks can be associated with high upfront costs. This may represent a significant barrier to uptake for many employers, despite some evidence to suggest these initial costs may be offset by reduced sickness and increased productivity in the future [23, 24]. Other intervention strategies such as educational or behavioural strategies or changes to organisational policies may not require the same level of financial investment, but have demonstrated inconsistent results [17, 18, 20].

The Medical Research Council (MRC) has highlighted the importance of understanding factors which may affect implementation of complex interventions [25]. Interventions may be ineffective due to poor design, but they may also be ineffective due to poor implementation. Thus, an intervention that has been developed and shown to be effective in one context, may not necessarily be effective when transferred to another. This may be particularly true for workplaces given the variations that exist in terms of size, sector, organisational structure and culture. In order to fully understand what works, it is essential to gain an understanding of factors that may influence implementation of “sit less” interventions in different workplaces. This information, in turn, would support the implementation of such interventions, allowing policy-makers and practitioners to determine whether an intervention will fit their specific context. To date, there are no systematic reviews exploring factors influencing the implementation of office-based sitting interventions. Therefore, this paper aims to: firstly, systematically review the literature to identify factors important for the implementation of interventions to reduce sitting time amongst office-based workers; and secondly, use these findings to develop an operational framework to support the future implementation of “sit less” interventions.

## Methods

A qualitative systematic review was undertaken. The review protocol was published via PROSPERO (ID: CRD42016052703), although there were some iterative alterations to the methods outlined in the protocol which are detailed in Additional File 1. The preferred reporting items for systematic reviews and meta-analyses (PRISMA) guidelines were used [26].

### Search strategy

Four electronic databases covering a wide range of relevant sources were searched in January 2017: Web of Science Core Collection; MEDLINE; PsycINFO; CINAHL. In addition, citation searches were carried out by examining the reference lists of relevant reviews to identify further studies. Grey literature was also searched for by reviewing Google, Google Scholar and Mendeley. The search strategy focussed on terms relating to employees and workplaces (population and setting), “sit less” interventions, and outcomes that reduced sedentary behaviour. The complete strategy is shown in Additional File 2.

**Fig. 1:**
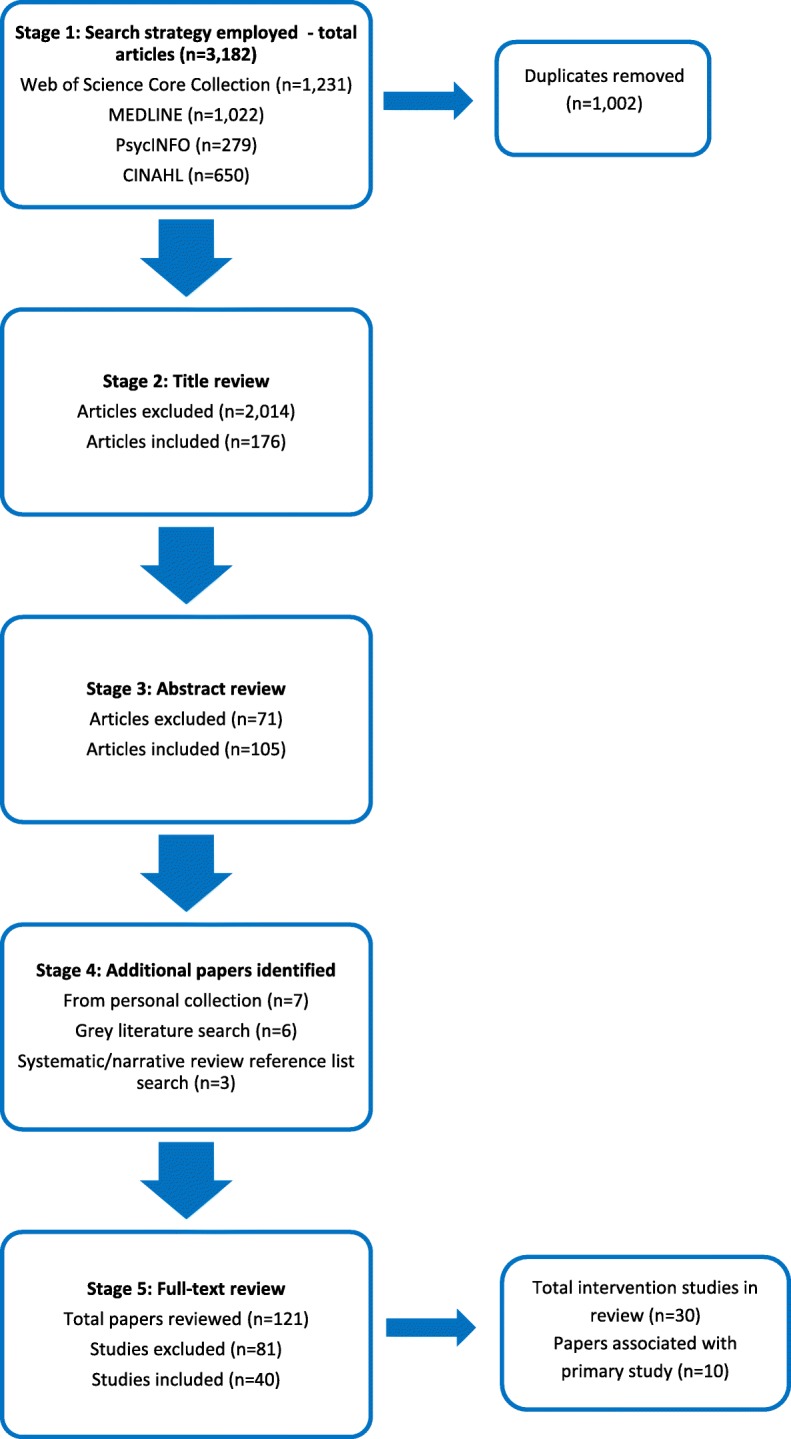
PRISMA flowchart of study selection process

Study selection was initially conducted by the lead author. This process involved five stages as outlined in Fig. 1. To ensure accuracy and consistency of study selection, a 20% random sample of the papers was independently checked by two additional reviewers at the title review stage. Disagreements were resolved by discussion between the reviewers until consensus was reached. Thereafter, the lead author determined study eligibility, but for those papers where the decision to include was unclear at the full-text review stage, a second reviewer independently checked papers and again, any disagreements were discussed until consensus was reached.

### Inclusion criteria

A broad range of study designs were required to provide rich data in terms of implementation factors from both experimental and observational studies. Study designs therefore included: randomised-controlled trials (RCTs), uncontrolled or controlled trials, before and after studies, and mixed-methods studies. Participants were employed adults in office-based jobs whose occupations involved spending the majority of their working time sitting, e.g., administration, customer service, help-desk professions, call-centre workers, receptionists, academics. Any intervention with the primary aim to reduce workplace sitting time and with a primary outcome of change in workplace sitting time was included. The primary outcome could be measured either objectively, e.g., accelerometer devices, or subjectively, e.g., self-report. In addition, papers associated with the primary study (“associated papers”), e.g., study protocols, intervention development papers and qualitative papers, were also included in the review.

### Data extraction and synthesis

A pre-piloted data extraction tool was developed to ensure consistent and rigorous data collection. For each included study, the lead author extracted descriptive data pertaining to: study design, setting (country and organisation type); participant information (sample size and demographic information); intervention description (duration, cost and the intervention development process); and measurement tools used. To further ensure accuracy and consistency of data extraction, a 10% random sample was coded by an additional reviewer. Data relating to intervention implementation came from either first-order constructs, i.e., direct participant quotes or findings reported by authors that were adequately supported by data from focus group discussions, interviews and surveys (where open question responses were recorded); or second-order constructs, i.e., author interpretations, statements, assumptions and ideas.

A formal assessment of quality of the included studies was conducted in order to potentially explain differences in results of otherwise similar studies (see Additional File 3). However, in line with the increasing understanding that qualitative data should be included in systematic reviews [27, 28], all studies were included in the review regardless of quality assessment. This was to allow the inclusion of qualitative data from a range of study designs that may provide a richer understanding of the factors important for the implementation of interventions to reduce workplace sitting.

A narrative synthesis of the descriptive data relating to study design, setting, participant information, intervention and outcome data was undertaken followed by an inductive thematic synthesis of the qualitative data to identify themes relating to intervention implementation. The synthesis followed the three stages as outlined by Thomas and Harden [27]: coding the text line-by-line, developing descriptive themes, and generating analytical themes. Codes were created inductively to capture the meaning and content of each extracted statement. The coding of the text allowed the translation of concepts from one study to another, but new codes continued to be added with each study that was analysed. Similarities and differences between the codes were then reviewed to allow grouping of the codes into a hierarchical structure where initial descriptive themes were identified. Thereafter, higher-order, analytical themes were generated.

## Results

Forty papers were included in the review, comprising 30 primary intervention studies [23, 29–57] and 10 papers that were associated with the primary intervention studies. These associated papers included two study protocols [58, 59], two intervention development papers [60, 61], three qualitative papers [62–64], one additional quantitative paper [65], one descriptive paper [66], and one paper published also as a dissertation report [67]. See Table 1 for an outline of the key characteristics of the primary studies and details of the associated papers and Additional File 4 for the extracted raw data.

**Table 1:** Study characteristics

Author (reference)	Study design	Setting	Participants	Intervention (description, complex or simple, duration, theoretical support)	Control group	Objective or subjective measure of sitting
Alkhajah et al. 2012 [29]	Non-randomised	Academic institution - health research, Australia	Total: *n* = 32 Intervention group *n* = 18 Control group *n* = 14	Sit-stand desk plus verbal and written instructions on best use (intervention duration: 3 months) - simple intervention Theory use not explicitly mentioned	Control group received no modifications	Objective (activPAL)
Chau et al. 2014 [40] *Associated paper (qualitative study):* *Chau* et al. *2014* [62]	Crossover RCT (with qualitative study embedded)	Non-government health agency, Australia	Total: *n* = 42	Sit-stand desk plus training on how to use and ergonomic assessment (intervention duration: 4 weeks) - simple intervention Theory use not explicitly mentioned	Control group received no modifications (remained on waitlist to receive intervention at the end of the study)	Objective (activPAL)
Chau et al. 2016 [51]	Non-randomised	Call centre, Australia	Total: *n* = 31 Intervention group *n* = 16 Control group *n* = 15	Sit-stand desk, brief training on use and daily email reminders to stand-up more during the first 2 weeks after installation (intervention duration: 19 weeks) - complex intervention Theory use not explicitly mentioned	Control group received no modifications	Objective (activPAL and ActiGraph) but low participant adherence so only presented subjective data (self-report) in paper (objective data was presented as supplemental information)
De Cocker et al. 2016 [53] *Associated paper: (intervention development)* *De Cocker* et al. *2015* [60]	RCT (2 interventions, one control)	University and environment agency, Belgium	Total: *n* = 213 Tailored group *n* = 78 Generic group *n* = 84 Control group *n* = 51	Web-based intervention - personalised computer-tailored advice with tips on how to reduce and interrupt sitting time (intervention duration not documented) - complex intervention Also generic intervention - non-personalised info on the importance of reducing/interrupting sitting time and tips on how to achieve this Theory used - theory of planned behaviour with the concept of goal-setting integrated (goal-setting and action plans operate within Self-Regulation Theory), also concepts of Self-Determination Theory	Control group received no modifications (remained on waitlist to receive intervention at the end of the study)	Objective (activPAL) but only a sub-sample (57%) used these, the rest were subjective (self-report)
Dutta et al. 2014 [54] *Associated paper (qualitative study): Dutta* et al. *2015* [63]	Crossover RCT with qualitative study embedded	Private sector organisation, USA	Total: *n* = 29 (*n* = 17 received intervention during period 1; *n* = 12 during period 2)	Sit-stand desks, advice on usage, email reminders to use desks (intervention duration: 4 weeks) - complex intervention Theory use not explicitly mentioned	Control group received no modifications	Objective (accelerometer - Modular Signal Recorder)
Evans et al. 2012 [55]	RCT	University, Scotland	Total: *n* = 30 Education only group *n* = 15 Point-of-choice prompts group *n* = 15	Education only - education session on adverse health effects of prolonged sitting Point-of-choice prompts - as above plus prompting software reminding them to stand every 30 mins - complex intervention (intervention duration: 5 days) Theory use not explicitly mentioned	Controls were the education only group	Objective (activPAL)
Gao et al. 2016 [56]	Non-randomised	University, possibly in Finland but not stated	Total: *n* = 92	Sit-stand desks (intervention duration: 6 months) - simple intervention but intervention participants also moved into a new building, so unclear if this contributed to changes seen Theory use not explicitly mentioned	Control group received no modifications	Subjective (self-report)
Gordon 2013 [57]	RCT	University, USA	Total: *n* = 24 Intervention group *n* = 13 Control group *n* = 11	Emails with psychosocial info and other available resources relating to decreasing SB at work (educational info, goal-setting, self-regulation, facilitation, reciprocal determinism (intervention duration: 10 weeks) - complex intervention All participants received walking workstation (intervention and control) Theory used - social cognitive theory	Control group received general health education - biweekly emails concerning general health topics frequently addressed in the workplace - educational materials were drawn from authoritative sources pertaining to that week’s topic	Objective (activPAL and ActiGraph)
Graves et al. 2015 [30]	Parallel-group RCT	University, England	Total: *n* = 44 Intervention group *n* = 23 Control group *n* = 21	Sit-stand desks, advice on usage (intervention duration: 8 weeks) - simple intervention Theory use not explicitly mentioned	Control group received no modifications	Subjective (ecological momentary assessment - EMA)
Healy et al. 2013 [23] *Associated paper (additional quantitative findings):* *Stephens* et al. *2014* [65]	Non-randomised	Government agency, Australia	Total: *n* = 43 Intervention group *n* = 22 Control group *n* = 21	Multicomponent intervention - organisational element (organisational strategies to sit less, liaison person in organisation), environmental element (sit-stand desks), individual element (health coaches with feedback) (intervention duration: approx. 4 weeks) - complex intervention Theory use not explicitly mentioned (although likely based on social cognitive theory and socio-ecological theory as per was a pilot for the study below)	Control group received no modifications	Objective (activPAL)
Healy et al. 2016 [31] *Associated papers:* *(protocol) Dunstan* et al. *2013* [59] *(intervention development) Neuhaus* et al. *2014* [61] *(pilot testing)* Healy et al. 2013 [23] and Neuhaus et al. 2014 [32] *(description paper) Healy* et al. *2016* [66]	Cluster RCT	Government agency, Australia	Total: *n* = 231 Intervention group *n* = 136 Control group *n* = 95	Multicomponent intervention - organisational element (organisational strategies to sit less, liaison person in organisation), environmental element (sit-stand desks), individual element (health coaches with feedback) (intervention duration: 12 months) - complex intervention Theory used - social cognitive theory and socio-ecological theory	Control group maintained usual practice but received written feedback on their activity and biomarker outcomes at 3-months (baseline and 3-month results provided) and 12-months	Objective (activPAL)
Neuhaus et al. 2014 [32]	Quasi-RCT	University, Australia	Total: *n* = 44 Multicomponent intervention *n* = 16 Workstation only *n* = 14 Control group *n* = 14	Multicomponent intervention - organisational elements (management support), environmental elements (sit-stand desks), individual elements (face-to-face coaching, feedback and goal-setting) - complex intervention Workstation only group too - simple intervention (intervention duration: 3 months) Theory used - social cognitive theory and socio-ecological theory	Control group received no modifications	Objective (activPAL)
Pronk et al. 2012 [33]	Non-randomised	Non-profit, health organisation, USA	Total: *n* = 34 Intervention group *n* = 24 Control group *n* = 10	Sit-stand desks as part of a comprehensive and multicomponent general health and wellbeing programme (intervention duration: 4 weeks) - simple intervention Theory use not explicitly mentioned	Control group received general health and wellbeing intervention but no sit-stand desks	Subjective (experience-sampling methodology) NB: Not used ESM score as don’t give a comparable measure of sitting time
Puig-Ribera et al. 2015 [34]	Quasi-RCT	4 x universities, Spain	Total: *n* = 264 Intervention group *n* = 129 Control group *n* = 135	Automated web-based program with range of ecological support strategies to facilitate decrease in sitting time (intervention duration: 19 weeks) - complex intervention Ramping phase - first 8 weeks; maintenance phase - 9-19 weeks; follow-up - 2 months after completion Theory use not explicitly mentioned	Control group received no modifications	Subjective (self-report)
Tobin et al. 2016 [35] *Associated paper (qualitative study):* *Leavy* et al. *2016* [64]	RCT (with associated qualitative study)	A non-government organisation (possibly private sector) and a university, Australia	Total: *n* = 37 Intervention group *n* = 18 Control group *n* = 19	Sit-stand desks plus info on usage and brief educational intervention (intervention duration: 4 weeks) - complex intervention Theory use not explicitly mentioned	Control group received no modifications	Objective (activPAL)
Urda et al. 2016 [36]	RCT	University, USA	Total: *n* = 44 Intervention group *n* = 22 Control group *n* = 22	Intervention: alert every hour to disrupt sitting, set in university scheduling system; also received handouts with ideas for light PA whilst at work and educational info (intervention duration: 1 week) - complex intervention Theory use not explicitly mentioned	Control group received no modifications	Objective (activPAL)
Brakenridge et al. 2016 [37] *Associated paper (protocol):* *Brakenridge* et al. *2016* [58]	Cluster RCT (2 interventions, no control)	Private sector organisation, Australia	Total: *n* = 153 Group ORG *n* = 87 Group ORG + tracker *n* = 66	Organisational support “Group ORG” - complex intervention including leaflets, emails, workplace champions, management support Group ORG + tracker - as above but with LUMOback device (belt that syncs with mobile app) which provides feedback on sitting time and activity (intervention duration: 12 months) Mention of socio-ecological model, but not confirmed that this was used in intervention development	Other intervention group (“Group ORG”) used as a comparator	Objective (activPAL)
Danquah et al. 2016 [52]	Cluster RCT	3 public sector and 1 private sector organisations, Denmark and Greenland	Total: *n* = 317 Intervention group *n* = 173 Control group *n* = 144	Multicomponent intervention - local ambassadors/ champions, management support, high meeting tables, routes for walking, educational lecture, workshop (strategies to reduce sitting developed), emails/text message reminders (intervention duration not documented) - complex intervention Sit-stand desks are standard in Denmark/Greenland, so all participants (intervention and control) had sit-stand desks. Theory used - social cognitive theory, Rogers’ diffusion on innovations theory and goal-setting theory	Other intervention group (with sit-stand desks provided as standard) used as comparator	Objective (ActiGraph)
Donath et al. 2015 [38]	RCT	Private sector health insurance company, Switzerland	Total: *n* = 31 Intervention group *n* = 15 Control group *n* = 16	Intervention group received sit-stand desks and also received pop-up messages to promote standing time (intervention duration: 12 weeks) - simple intervention Theory use not explicitly mentioned	Other intervention group (with sit-stand desks provided as standard) used as comparator	Objective (ActiGraph)
Gilson et al. 2016 [39]	Non-randomised	Tele-communications, Australia	Total: *n* = 57 Intervention group 1 *n* = 33 Intervention group 2 *n* = 24	Intervention 1: Co-produced intervention with a range of strategies to sit less - complex intervention Intervention 2: as above plus real-time feedback and prompts to sit less - complex intervention (intervention duration: 5 months) Theory use not explicitly mentioned	Other intervention group (“intervention 1”) used as comparator	Objective (sitting pad)
Swartz et al. 2014 [41]	Parallel-group RCT	University, USA	Total: *n* = 68 Stand group *n* = 38 Step group *n* = 30	Wrist-worn prompt to disrupt 60 continuous minutes of SB Stand group - get up from their chairs when prompt went off Step group - do 100 steps when prompted (intervention duration:? 1 week - unclear) Simple intervention Theory use not explicitly mentioned	Other intervention group (“Step group”) used as comparator	Objective (activPAL)
Gilson et al. 2012 [42]	Pre-post intervention	Open plan office, not clear which type of organisation, Australia	Total: *n* = 11	Sit-stand desks, educational brief re. benefits of reducing sitting time (intervention duration: 1 week) - complex intervention Theory use not explicitly mentioned	No control group	Objective (wrist accelerometer)
Gorman et al. 2013 [43] *Associated paper (dissertation report): Gorman 2012* [67]	Pre-post intervention - natural experiment	Academic physical activity research centre Canada	Total: *n* = 24	Intervention: Move to purpose-built office space (specifically designed by research group) activity permissive physical environment (included sit-stand desks) (intervention duration: 3 months) - complex intervention but single level of influence (environmental only) Theory use not explicitly mentioned	No control group	Objective (activPAL)
Grunseit et al. 2013 [44]	Mixed methods - pre-post in natural setting + qualitative study	Government organisation, Australia	Total: *n* = 18	Sit-stand desks (permanent intervention, but post measures done after 92 days) - simple intervention Theory use not explicitly mentioned	No control group	Subjective (self-report)
Jancey et al. 2016 [45]	Pre-post intervention -natural study	Unclear if private sector business organisation, Australia	Total: *n* = 42	Intervention: move to a purpose-built building that was activity-permissive (permanent intervention, but post measures done at 4 months) - single level intervention (environmental) but complex given nature of a building move Theory use not explicitly mentioned	No control group	Objective (ActiGraph)
Mackenzie et al. 2015 [46]	Pre-post intervention	Health-related research university, England	Total: *n* = 26	Multicomponent intervention with management support, prompts, educational element, use of social media (co-produced intervention) (intervention duration: 4 weeks) - complex intervention Theory used - socio-ecological model	No control group	Subjective (self-report)
Mansoubi et al. 2016 [47]	Pre-post intervention	University, England	Total: *n* = 40	Sit-stand desks plus educational element plus online planning tool for comfortable computing (intervention duration: 3 months) - complex intervention Theory use not explicitly mentioned	No control group	Objective (activPAL and ActiGraph)
Parry et al. 2013 [48]	Parallel-arms cluster RCT	3 x Government organisations, Australia	Total: *n* = 133 Intervention A *n* = 49 Intervention B *n* = 30 Intervention C *n* = 54	Intervention A: active office work (daily access to height-adjustable desk with integrated treadmill, or a treadmill plus a stationary cycle ergometer, plus other suggestions for staff to be actively working) Intervention B: traditional PA (pedometer challenge, active transport, active work, lunchtime walks) Intervention C: office ergonomics (active sitting, standing meetings, use of piano stool / air cushion) (intervention duration 12 weeks) NB some of the intervention elements were common to different groups All complex interventions Theory use not explicitly mentioned	No “no intervention” group	Objective (ActiGraph)
Priebe et al. 2015 [49]	RCT	Private sector organisation, unclear of country setting, possibly Canada	Total: *n* = 99 HP/HC group *n* = 23 HP/LC group *n* = 24 LP/HC group *n* = 25 LP/LC group *n* = 27	Email messages - received 1 of 4 different types: - high personal/high contextual (HP/HC) - high personal/low contextual (HP/LC) - low personal/high contextual (LP/HC) - low personal/low contextual (LP/LC) Complex intervention - only email message but personalised and contextualised One email and follow-up immediately and 3 work days after (intervention duration: 1 day) Theory used - focus theory (descriptive norms)	No “no intervention” group	Subjective (self-report)
Richards and Brain 2015 [50]	Pre-post intervention	University, Wales	Total: *n* = 18	Multicomponent intervention - began with a one-day event (On your feet Britain (OYFB)), then 30 min presentation identifying strategies to reduce sitting, email reminders daily, OYFB posters/leaflets (intervention duration: 10 days) - complex intervention Theory used – Behaviour Change Wheel, Theoretical Domains Framework, COM-B model, Theory of Planned behaviour	No control group	Subjective (self-report)

### Study design and setting

The 30 primary intervention studies comprised 17 RCTs [30–32, 34–38, 40, 41, 48, 49, 52–55, 57], six non-randomised trials [23, 29, 33, 39, 51, 56], six pre-post intervention studies [42, 43, 45–47, 50], and one mixed-methods study [44].

Thirteen of the intervention studies were set in Australia [23, 29, 31, 32, 35, 37, 39, 40, 42, 44, 45, 48, 51], six in the United States or Canada [33, 36, 41, 43, 54, 57], five in the United Kingdom [30, 46, 47, 50, 55], four in mainland Europe [34, 38, 52, 53], and two did not explicitly state the country setting [49, 56]. Fifteen studies were conducted either solely [29, 30, 32, 34, 36, 41, 43, 46, 47, 50, 55–57], or in part [35, 53], within an academic institution. The remaining studies were conducted within a range of other public, private and voluntary sector organisations.

### Sample size and participant characteristics

The combined population of the 30 intervention studies included in this review was 2271 participants, with the total sample size per study ranging from 11 to 317. All except three of the intervention studies [37, 39, 51] were female-dominated, ranging from 53 to 100% of samples. Nineteen of the studies [23, 29, 30, 32, 33, 35, 37, 40, 41, 43–46, 50, 52, 53, 55–57] had majority participants with tertiary-level education or who were in professional (not administrative) job roles.

### Interventions

Intervention duration ranged from one day to 12 months. Three studies did not clearly document intervention duration [41, 52, 53]. Two natural experiments [44, 45] involved the evaluation of permanent interventions.

The majority of studies evaluated interventions which involved the use of ergonomic interventions such as sit-stand desks or height-adjustable treadmill desks, either alone [29, 30, 40, 44, 56] or in combination with other intervention components such as the provision of educational information [35, 42, 47, 51, 52], prompts [38, 51, 52, 54], promotion of other “sit less” initiatives, e.g. walk and talk meetings [48], the use of health coaches/workplace champions [23, 31, 32, 52], individualised feedback [23, 31, 32], or as part of a wider health and wellbeing programme [33]. Other interventions included: automated web-based programmes with a range of support strategies [34, 53]; use of a wrist-worn device to disrupt sedentary behaviour [41]; environmental adaptation via a move to a new purpose-built building [45]; and multi-component interventions using a variety of low-cost strategies, e.g. leaflets, posters, emails, prompts, workplace champions and management support to encourage staff to reduce workplace sitting [36, 37, 39, 46, 49, 50, 55, 57].

### Outcome measures

Twenty-two studies used an objective measure of sitting time via inclinometers (activPAL3) [23, 29, 31, 32, 35–37, 40, 41, 43, 47, 51, 53, 55, 57], and/or accelerometers (e.g., ActiGraph) [38, 42, 45, 47, 48, 52, 54, 57], or a sitting-pad device [39]. The remaining studies used subjective measures of sitting time via self-report/questionnaires [34, 44, 46, 49, 50, 56], ecological momentary assessment (e.g., using a paper diary to report whether they were sitting, standing or walking every 15 min) [30], or experience-sampling methodology (e.g., responding to a text message sent three times per day) [33].

### Thematic synthesis

Qualitative data relating to factors affecting intervention implementation were extracted from 34 of the 40 papers. The majority of the qualitative data extracted came from second-order constructs (author interpretations, statements, assumptions and ideas) from the following associated papers: three qualitative papers [62–64], one intervention development paper [61], one descriptive paper [66], one mixed-methods paper [44], and one dissertation report [50]. Other papers provided smaller amounts of useful qualitative data [23, 30–34, 36–40, 42, 43, 45–49, 51–54, 57, 59, 60, 65, 67].

The thematic synthesis coding process created 40 initial codes, from which emerged nine descriptive themes. Three higher-order analytic themes were then derived from these nine descriptive themes (see Table 2). Some descriptive themes were found to cut across several analytical domains.

**Table 2:** Descriptive and analytic themes

	Analytic Themes		
Descriptive Themes	Intervention Development	Intervention Implementation	Intervention Evaluation
Understanding local barriers and facilitators to participation	X		
Identifying and using a theoretical model to operationalise intervention strategies	X		
Using participatory or collaborative approaches	X	X	
Conducting a pilot study within the target organisation	X	X	
Developing and implementing an action plan incorporating key intervention characteristics	X	X	
Embedding the intervention within local policy strategies or high-level management		X	
Conducting a comprehensive process evaluation			X
Conducting an outcome evaluation involving a range of measures			X
Taking into account the “real-world” context	X	X	X

### Understanding local barriers and facilitators to participation

Findings highlighted the importance of identifying and understanding local barriers and facilitators to intervention participation during the development phase. Individual-level barriers to interventions included: individual preference for a seated working style [44, 62, 67]; feelings of self-consciousness when standing due to the perception of being a distraction to seated colleagues [30, 50, 60, 62, 64]; the perception from staff and/or managers that sitting less initiatives could negatively impact on productivity [44, 50, 60, 62]; and work-related factors such as the nature of work, workload and time [30, 46, 50, 60, 62]. The idea that sitting in the workplace represents a social norm [50, 62] and the issue of the physical work environment providing limited opportunities to sit less [37, 38, 44, 46, 50] were identified as further barriers. Some papers noted cost as a potential barrier to large scale roll-out, given the high upfront costs for interventions that included sit-stand desks [40, 44, 51, 64] or larger-scale environmental changes [43]. Several facilitators to “sit less” interventions were also highlighted including: perceived benefits to physical health, stress levels and productivity [44, 62]; and perceptions of peer [30, 44, 50, 64] and/or management support [23, 30, 31, 33, 36, 40, 51, 57].

### Identifying and using a theoretical model to operationalise intervention strategies

Identifying and using one or more behaviour change theoretical model was found to be an important step during the development of intervention strategies. Nine studies reported the use of a theoretical model to inform intervention design. Single theories were used in four studies including: Social Ecological Model (SEM) [46, 58], focus theory [49], and Social Cognitive Theory (SCT) [57]. Combined theories were reported in five studies: SCT, Rogers’ Diffusion on Innovations Theory and Goal-Setting Theory [52]; SCT and SEM [31, 32]; Theory of Planned Behaviour (TPB), with supporting concepts from both Self-Regulation Theory and Self-Determination Theory [53]; and the COM-B model (using the Behaviour Change Wheel and Theoretical Domains Framework) and TPB [50]. The use of theory ranged widely from: a simple mention of a theoretical model [37]; to describing the use of theory to support the development of interventions [31, 32]; to using theory to support intervention development and identify specific theoretical constructs and how these were operationalised [46, 53, 60]; and finally to using theory to support intervention development, implementation and process evaluation [49, 50, 52, 57]. Grounding intervention development within one or more theoretical models was believed by some of the included studies and/or associated papers to enhance intervention effectiveness [46, 49, 52, 53, 57, 61].

### Using participatory or collaborative approaches

It was reported that employee participation with development and implementation ensured that the intervention was acceptable and feasible for employees [46], supported engagement, tailoring and buy-in for the intervention [48], and highlighted and proposed ways to overcome anticipated barriers to intervention implementation [60]. Ten of the papers explicitly reported or recommended the use of participatory approaches during intervention development and/or implementation. Eight of these studies reported or recommended participation either via top-down involvement of team leaders/management [51], or bottom-up discussions with workplace champions [37] and/or groups of employees [23, 39, 46, 48, 52, 66] as part of workshops, focus groups, a workplace wellbeing committee, and information and consultation sessions. Two papers [61, 62] reported the use of collaborative approaches where both managers and employees were involved in intervention implementation.

### Conducting a pilot study within the target organisation

Three of the studies in this review were pilot studies [23, 32, 46], acting as precursors to larger trials. Two other papers described the use of pilot testing as part of the intervention development and/or implementation [52, 61]. The latter two papers reported the benefits of pilot testing to include: establishing what facilities were available in the workplace; understanding routines, interactions between employees and meeting frequencies; determining intervention efficacy, acceptability and feasibility; and allowing time for testing the implementation of the various intervention components. This information provided an opportunity to refine the intervention based on feedback from participants. Conducting a pilot within the organisation of interest therefore was anticipated to maximise the effectiveness of the intervention.

### Developing and implementing an action plan incorporating key intervention characteristics

Developing and implementing an action plan [66] which incorporates a range of key intervention characteristics was an important finding. Key characteristics that were identified included:

- Tailoring an intervention to ensure that it meets the needs of individuals and/or the organisation and is relevant to different groups of employees within an organisation [32, 34, 38, 48]. Tailoring may be supported by the use of theoretical models [32, 60] and a participatory approach to intervention development [31, 39, 46, 48].
- Having a menu of strategies to provide more choice for both employees and employers [62], which can be developed using participatory approaches tailored to specific organisational contexts [39].
- Using multi-component intervention to target multiple levels of influence, i.e., at the individual-, social-, organisational- and environmental-level was characteristic [23, 31, 32, 38, 39, 46, 52] and reported to have the potential for a more comprehensive and sustainable change on workplace sitting time compared to single-level interventions [33, 39, 43, 57, 61].
- Involving workplace champions in the development and/or implementation of an intervention was found to be a beneficial strategy [23, 31, 37, 46, 61]. Workplace champions were reported to promote intervention messages and create a supportive culture within the organisation to aid change [66] or be agents of change, to advocate for the allocation of resources and influence organisational policy targeting workplace sitting [64].
- Ensuring interventions are low-cost which reflects the finding described above relating to cost being a barrier to intervention uptake [32, 40, 51, 62, 64].
- Considering interrupting versus replacing sitting time, as one study found that interrupting workplace sitting may be more feasible that replacing longer periods of sitting with standing [60].

### Embedding the intervention within local policy strategies or high-level management

For effective intervention implementation and organisational change, some studies reported ensuring the intervention was supported by management and aligned with the target organisation’s policies and/or strategies. This was achieved using one or more of the following initiatives: engaging management and gaining their commitment for the intervention [23, 46, 51, 59, 61, 62]; identifying and understanding an organisation’s priorities or image by obtaining a clear description of the organisation’s processes and structures that may relate to intervention implementation [51, 62]; and where possible, embedding the intervention within the organisation’s processes, structures, policies and/or strategies [23, 38, 43, 48, 61].

Methods for engaging management reported by studies in this review were wide-ranging and included: managers being responsible for explicitly promoting the intervention [62]; using managers from relevant departments to facilitate the logistics of implementing an intervention, e.g., risk manager monitored planning and implementation, health and wellness manager co-ordinated all parties and arranged for the researchers to gain access to required local data [51]; presenting a business case to managers and gaining formalised commitment to the intervention [64, 66]; asking managers to distribute emails relating to the intervention [23, 39, 46]; and gaining managers’ consent for their staff to participate in the intervention [23, 30–32]. Consultation with managers also allowed the identification of organisational processes and structures that may be important in terms of intervention implementation [61]. These included information and potential changes in: job design, the physical work environment, workplace social norms, or workplace culture [23]. However, one study identified difficulties in changing organisational culture and suggested the need for stronger external support, such as the use of guidelines, as a way to support this change [48].

### Conducting a comprehensive process evaluation

No studies explicitly mentioned the use of process evaluation, but it was undertaken to some degree by seven studies [31, 32, 37, 46, 50, 52, 62]. Process evaluation either encompassed an assessment of intervention feasibility and acceptability and/or a determination of intervention fidelity. Feasibility and acceptability was generally assessed using qualitative methods, e.g., focus groups [32, 46, 50]. Intervention fidelity used quantitative measures, e.g., surveys, aiming to establish: the “dose” of intervention delivered and whether it was implemented as planned; the “dose” that was received; and whether there were any harms or unintended consequences associated with the intervention [31, 32, 37, 52]. In addition, three studies explored the mechanisms of change by considering personal and organisational motivations which led to initial and continued participation in “sit less” interventions [44, 62, 64]. Examples of these motivations included: curiosity to try something new; interest in potential health benefits and/or experiencing changes in health outcomes; perceived improvements in productivity and energy levels; personal challenge; relevance to employees’ organisation’s priorities; developing task- and time-based routines, e.g., certain tasks were easier to undertake whilst not sitting and time acted as a trigger/prompt to sit less; and an awareness of the issues associated with prolonged sitting at work, which led to a shift in the perspectives of peers or managers and/or a change in organisational culture, providing employees with informal “permission” to sit less at work.

### Conducting an outcome evaluation involving a range of measures

Measuring sitting/standing/moving time both within and outside of the workplace was believed to be important for intervention evaluation due to the possibility of a compensation effect, e.g., a reduction in sitting time at work resulting in an increase in sitting time at home [47, 67]. Addressing wider reaching outcomes was undertaken by some studies in this review to support a greater understanding of additional impacts of workplace sitting interventions. These included: the impact on physical (primarily musculoskeletal effects) [33, 46, 50, 62–64] and mental health and wellbeing [46, 50, 64]; work-related factors such as changes to productivity, alertness and concentration [30, 46, 50, 57, 62–64, 67]; staff morale and autonomy with feelings of empowerment to change workplace sitting behaviour [62, 64]; and wider socio-environmental changes or shifts in organisational culture [23, 30, 46, 50, 62, 63]. Finally, including a measure of intervention cost in order to allow an assessment of return on investment, which balances costs with potential productivity trade-offs, was found to be a potential facilitator to intervention uptake and therefore an important outcome to evaluate [51]. However, none of the studies reported formal cost-effectiveness data. Four papers briefly reported the costs for a single sit-stand desk as US$400–900 [54], £360–375 [30], US$400 [23], US$499 [32]. Eight papers identified that the intervention was “low-cost” [31, 34, 46, 49, 50], “a low resource intensive intervention” [37] or less expensive than more resource intensive individual-level interventions [38, 52], without presenting any quantitative cost data.

### Taking into account the “real-world” context

There was a paucity of contextual information reported in the included studies. Most of the studies presented information on the type of organisation(s) participating in the study and the sector within which that organisation was based. Only a few studies reported additional contextual factors such as information of different occupational roles or tasks [39, 48, 52], the organisation’s prior interest/involvement in workplace health initiatives [33, 37], local organisation processes [48], and the physical work environment [52, 67]. Typically, this contextual information was only briefly described. Two papers provided some in-depth contextual information relating to the varying job roles, local organisation processes and expectations of employees [48], and the physical environments of participating workplaces [67]. However, no study explicitly included information on the hierarchical structure, the organisational culture, and the wider political and economic landscape. Therefore, it was not possible to gain a clear picture of the contexts within which the interventions were developed, implemented and evaluated.

### Operational intervention framework

**Fig. 2:**
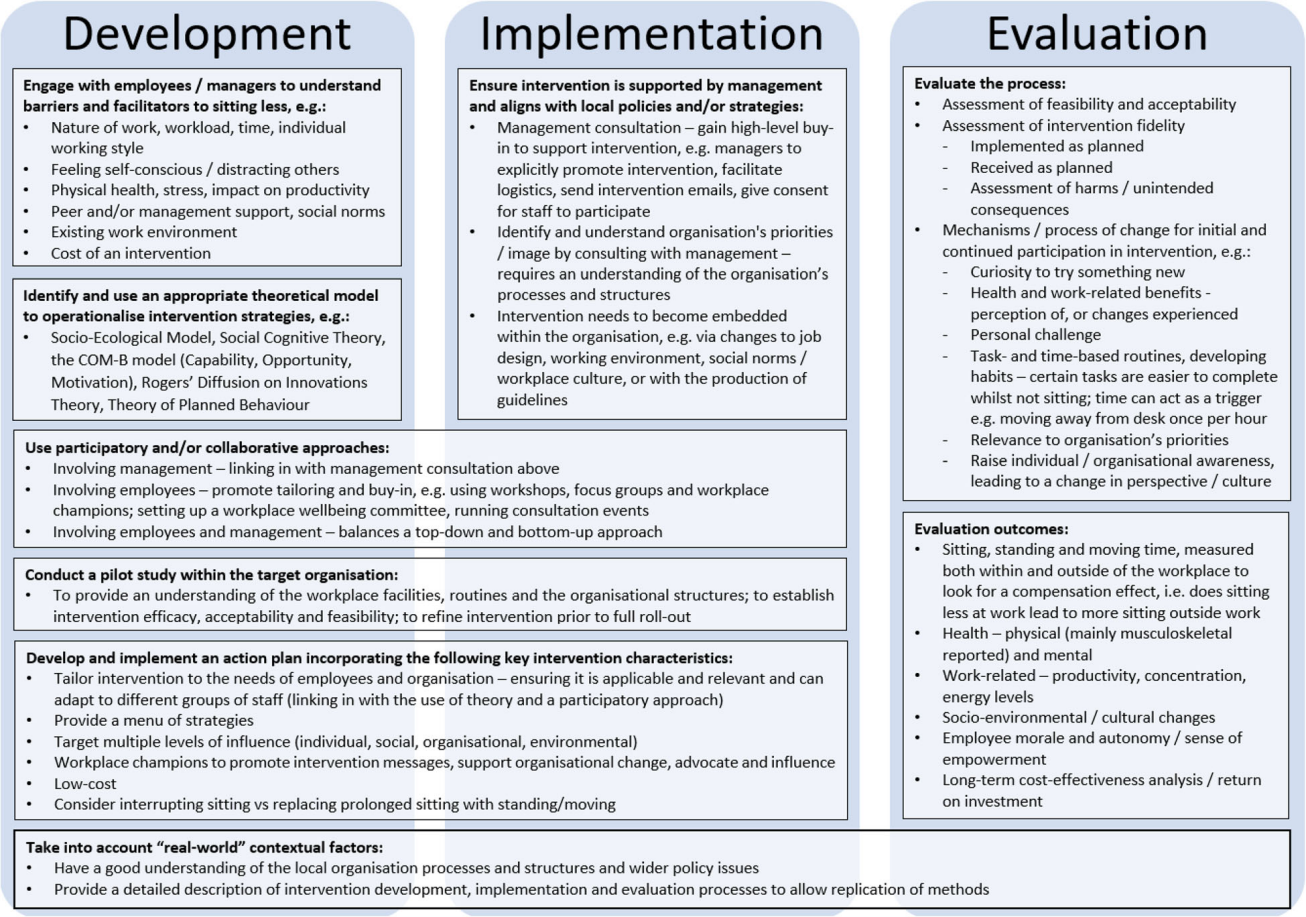
“Sit less” intervention development, implementation and evaluation operational framework

The findings of this review were translated into an operational framework to guide the future development, implementation and evaluation of interventions to reduce workplace sitting time (see Fig. 2). This framework displays the higher-order analytical themes within three large boxes labelled as *development*, *implementation* and *evaluation*. The associated lower-order descriptive themes were translated into strategies to overcome the issues or to incorporate elements of good practice as highlighted above.

## Discussion

The first aim of this systematic review was to identify key considerations for the implementation of interventions to reduce workplace sitting time. Based on the qualitative evidence synthesis, three higher-order themes relating to the *development*, *implementation* and *evaluation* of “sit less” interventions, and nine associated lower-order descriptive themes emerged. Many of the lower-order descriptive themes align with strategies identified by the MRC for the development and evaluation of complex interventions [68]. For example, exploring barriers and facilitators to intervention development, involving stakeholders/users during intervention development and implementation, conducting a pilot study, undertaking a thorough a process and outcome evaluation and understanding contextual factors [68]. In addition, MRC guidance recommends the use of a theory-driven approach during the development of complex behaviour change interventions [68]. However, this review highlighted the general lack of use, or detailed reporting of, a theoretical underpinning during intervention development, with only nine of the studies reviewed addressing theoretical constructs. Further, these studies used a range of theories with no single theory appearing dominant, although it is notable that the theories largely drew from the psychological and behavioural sciences. The socially-situated nature of sedentary behaviour also provides an opportunity for the use of more sociological or organisational cultural approaches [69]. Despite the appearance of descriptive themes consistent with MRC guidance, they were only reported by a minority of studies.

The second aim was to develop an operational framework which may help to facilitate a more structured approach to developing, implementing and evaluating workplace sitting interventions and ensure consideration is given to all of the nine descriptive themes. This framework not only summarises the findings of this review, but also represents an original contribution to the knowledge base, which now requires testing in order to refine and build on this initial version. It is possible that there may be themes missing from this analysis and the operational framework. This was demonstrated by the under-reporting of contextual and process-oriented factors. Contextual factors which may be important include details of local organisation processes and structures, e.g., size of organisation, sector, hierarchical structure and organisational culture, as well as the wider political and economic landscape [69]. A recent paper looking at the social ecological correlates of objectively measured workplace sedentary behaviour found that there are work-specific individual, cultural, environmental and organisational factors associated with sedentary behaviours in the workplace and that these associations vary by job type and sector [70]. Details relating to local policy, the wider political landscape, economic issues, the physical environment, and hierarchical structures and organisational culture, would all make it possible to understand and evaluate how the intervention effects may be impacted by one, or a combination, of these factors [71, 72]. Furthermore, the readiness for change of the organisation, and the quality of existing policies and practices employed by the organisation are all key functions of effective intervention implementation [72]. It is possible that workplace contextual factors were considered during intervention development and implementation but simply not reported in some studies.

Having a detailed understanding of the context will support effective intervention implementation, a pre-requisite for effectiveness [72] and, if comprehensively reported, will aid knowledge translation into “real-world” settings. Therefore, at present, it is intended that the operational framework developed in this review be used by researchers as a prompt to ensure considerations are explicitly given to the key factors of intervention development, implementation and evaluation, which is then comprehensively reported. Once tested and refined, the framework will then be translated into as a practical tool for organisations to guide their own “sit less” intervention development, implementation and evaluation.

Most of the studies included in the review were RCTs and used objective measurement tools (e.g., activPAL inclinometers). This represents an improvement in study design compared to previous reviews [17, 20]. However, this review demonstrates the importance of gathering context-rich and process-oriented data to more fully understand the factors that may promote or impede intervention effectiveness. The participants of the included studies were mainly well-educated females. This may be due to the most common setting for these studies being within academic institutions which employ large numbers of tertiary educated staff and because physical activity interventions tend to attract mainly female participants [73]. Therefore, future research should seek to develop and evaluate workplace sitting interventions outside of academic institutions.

The current review has a number of strengths. First, this review is the first to undertake a qualitative assessment of interventions to reduce workplace sitting. Second, due to the need to obtain rich qualitative data, a wide range of studies were included, allowing a broad understanding of factors relating to the development, implementation and evaluation of interventions aimed at reducing workplace sitting time. Third, the qualitative evidence synthesis provided the basis for the development of an evidence-based operational framework, which after a period of testing and refinement, could be translated into a practical toolkit for use by a range of organisations, thereby supporting knowledge mobilisation into “real-world” settings.

The review findings should also be considered in the context of several limitations. First, the search was limited to only four databases and, as a result, some studies may have been missed. Second, articles were limited to those published in English, which may have resulted in relevant articles published in other languages being missed. However, to ensure all relevant papers were identified, the lead author conducted a citation search of all systematic reviews that synthesised intervention studies with similar inclusion/exclusion criteria. The citation search did not identify any non-English intervention studies. Third, the qualitative evidence synthesis and subsequent framework development is limited to the qualitative data within the published papers. There were papers where no qualitative data were extracted due to a lack of reporting of factors relating to the implementation of interventions. It is likely that there are insights from these studies that could also be used to further inform the framework. Future research should aim to provide more detailed reporting of factors relating to intervention development, implementation and evaluation.

## Conclusions

This qualitative systematic review explored factors that aid the effective implementation of workplace sitting interventions. The findings indicate a need for comprehensive intervention development, implementation and evaluation processes which should focus on a range of strategies including: understanding the barriers and facilitators to participating in workplace sitting interventions; identification and use of a theoretical model; gaining management support and ensuring an intervention aligns with existing policies/strategies; the use of participatory approaches; conducting a pilot study; developing and implementing an action plan; and undertaking a comprehensive process and outcome evaluation. In addition, an under-reported cross-cutting consideration relates to the consideration of the context within which interventions are undertaken. The qualitative synthesis findings led to an operational framework to inform the planning of future “sit less” interventions. This framework needs to be formally tested in a range of workplace settings to establish whether it is fit for purpose and whether it adequately captures all relevant contextual factors. This work, in turn, will help to ensure that the potential of the framework to inform the development, implementation and evaluation of effective interventions to reduce workplace sitting time is realised.

## Additional files

Additional File 1: Amendments to the Original PROSPERO Protocol. (DOCX 14 kb)

Additional File 2: Search Strategy. (DOCX 14 kb)

Additional File 3: Quality Assessment. (DOCX 67 kb)

Additional File 4: Raw Qualitative Data. (DOCX 46 kb)

## Abbreviations

COM-B: Capability Opportunity Motivation Behaviour Change Model
MRC: Medical Research Council
PRISMA: Preferred Reporting Items for Systematic reviews and Meta-analyses
RCT: Randomised-Controlled Trials
SCT: Social Cognitive Theory
SEM: Social Ecological Model
TPB: Theory of Planned Behaviour
