# Supplementary material for: The development, implementation and evaluation of interventions to reduce workplace sitting: a qualitative systematic review and evidence-based operational framework
Source: BMC Public Health. 2018 Jul 4;18:833. doi: 10.1186/s12889-018-5768-z (PMC6033205; doi:10.1186/s12889-018-5768-z)
Supplement: Supplementary file 1 — Amendments to the Original PROSPERO Protocol. (DOCX 14 kb) [file 12889_2018_5768_MOESM1_ESM.docx]

**Additional File 1: Amendments to the Original PROSPERO Protocol**

**Initial Search Strategy**

An initial search was conducted as per the PROSPERO protocol. The results were sifted by title and then by abstract. However, after cross-referencing papers in the lead author’s personal collection that met the inclusion criteria based on abstract, this initial search strategy was deemed not to be sensitive or specific enough. More than 40 potentially relevant studies that were in the lead author’s personal collection had not been picked up by the initial search strategy. Therefore, a second search strategy was devised as described in the paper and detailed further in Additional File 2.

This revised search strategy was simplified and removed the MeSH terms and the “Barriers, levers, facilitators, mediators, moderators or factors” search terms. It was determined that the terms relating to the implementation factors would be better elicited from the data extraction process, as there may be contextual factors that are not highlighted by the authors as barriers or facilitators, but which may appear to be relevant, e.g., type of organisation. In addition, a “NOT” search criterion was included relating to children and adolescents, as the original search had identified many papers involving children or schools which were not relevant. Filters within the databases were set to select only English language papers, with no limit to the date of publication.

**Quality Assessment**

In the protocol, it was anticipated that that Critical Appraisal Skills Programme (CASP) checklists would be used for assessing the quality of each of the papers to be included in the review as the CASP checklist covers a wide range of study designs. However, after discussion with an expert in synthesis methods it was determined that the more appropriate method of quality assessment for this review would be to utilise the best tool available for each study type rather than one generic tool. Those tools are described in Additional File 3.
