# Supplementary material for: The development, implementation and evaluation of interventions to reduce workplace sitting: a qualitative systematic review and evidence-based operational framework
Source: BMC Public Health. 2018 Jul 4;18:833. doi: 10.1186/s12889-018-5768-z (PMC6033205; doi:10.1186/s12889-018-5768-z)
Supplement: Supplementary file 2 — Search Strategy. (DOCX 14 kb) [file 12889_2018_5768_MOESM2_ESM.docx]

**Additional File 2: Search Strategy**

The search strategy was conducted as follows:

|  | **Terms** |
| --- | --- |
| **1. Population/setting** | ((Employee* **OR** staff **OR** workforce **OR** worker* **OR** occupation* **OR** workplace* **OR** desk* **OR** office*) **NOT** (Child* **OR** adolescen* **OR** school*)) |
| **2. Intervention** | (Education **OR** counselling **OR** prompt* **OR** reminder* **OR** management support **OR** sit-stand **OR** stand* **OR** workstation*) |
| **Comparators** | *Not applicable* |
| **3. Outcomes** | (Sedentary **OR** sitting **OR** inactivity) |
| **Study design** | *Not applicable* |

Search Limits

| **Study designs** | None |
| --- | --- |
| **Publication types** | None |
| **Date of publication** | None |
| **Language** | English |
| **Other limits** | None |

Sources to be searched

- Medline, PsycINFO, CINAHL, Web of Science
- Check reference lists of all relevant papers
- Search websites for grey literature, e.g., Google, Google Scholar and Mendeley.
