# Supplementary material for: The development, implementation and evaluation of interventions to reduce workplace sitting: a qualitative systematic review and evidence-based operational framework
Source: BMC Public Health. 2018 Jul 4;18:833. doi: 10.1186/s12889-018-5768-z (PMC6033205; doi:10.1186/s12889-018-5768-z)
Supplement: Supplementary file 3 — Quality Assessment. (DOCX 67 kb) [file 12889_2018_5768_MOESM3_ESM.docx]

**Additional File 3: Quality Assessment**

In order to assess the quality of the range of study designs included in this review, the most appropriate tool for each study design was used to assess quality which included: Cochrane Risk of Bias Tool (1) for randomised controlled trials (RCTs) (including crossover trials and cluster RCTs); Risk of Bias in Non-Randomised Studies-of Interventions (2) for non-randomised trials; Mixed-Methods Appraisal Tool (3) for mixed methods studies; and the Critical Appraisal Skills Programme (CASP) Tool (4) for qualitative studies. Quality assessment was not formally undertaken for pre-post intervention studies, as this study design inherently encompasses high levels of bias which cannot be mitigated. Instead all pre-post intervention studies were categorised as high-risk. However, given the increasing understanding that qualitative data should not be excluded (5,6) and since it was anticipated that qualitative data would be extracted from all studies in this review, no studies were excluded based on the quality assessment. Instead, an assessment of quality was conducted in order to potentially explain differences in results of otherwise similar studies.

Qualitative studies which were linked to intervention studies but published in a separate paper (7–9), were quality assessed separately using the CASP tool. Therefore, a total of 27 papers underwent formal quality assessment, details of which can be seen in Tables 3.1 to 3.4.

*Table 3.1: Summary quality assessment of RCTs (Cochrane Risk of Bias Tool)*

| Author (reference) | Random sequence generation | Allocation concealment | Blinding of participants and personnel | Blinding of outcome assessment | Incomplete outcome data | Selective reporting | Other sources of bias | Overall assessment |
| --- | --- | --- | --- | --- | --- | --- | --- | --- |
| Brackenridge et al. (10) | + | ? | X | ? | + | + | ? | X |
| Chau et al. (11) | + | ? | X | ? | + | X | ? | X |
| Danquah et al. (12) | + | ? | X | + | + | ? | ? | X |
| De Cocker et al. (13) | ? | + | X | ? | ? | ? | X | X |
| Donath et al. (14) | + | ? | X | ? | ? | ? | ? | X |
| Dutta et al. (15) | + | ? | X | ? | + | ? | ? | X |
| Evans et al. (16) | + | + | X | + | + | ? | + | X |
| Graves et al. (17) | + | + | X | X | + | ? | X | X |
| Healy et al. (18) | + | ? | X | X | + | X | + | X |
| Neuhaus et al. (19) | ? | + | X | X | ? | ? | X | X |
| Parry et al. (20) | + | ? | X | X | X | ? | ? | X |
| Puig-Ribera et al. (21) | ? | + | X | ? | + | ? | X | X |
| Swartz et al. (22) | + | ? | X | ? | ? | ? | ? | X |
| Urda et al. (23) | + | + | X | X | ? | ? | ? | X |
| Priebe et al. (24) | + | + | X | ? | ? | ? | X | X |
| Gordon (25) | ? | ? | X | ? | + | ? | + | X |
| Tobin et al. (26) | ? | ? | X | ? | ? | ? | ? | X |

*+, low-risk of bias; X, high-risk of bias; ?, unclear-risk of bias*

*Table 3.2: Summary quality assessment of non-randomised trials (Risk of Bias in Non-Randomised Studies-of Interventions)*

| Author (reference) | Confounding | Selection of participants | Classification of interventions | Deviations from intended interventions | Missing data | Measurement outcomes | Selection of reported results | Overall assessment |
| --- | --- | --- | --- | --- | --- | --- | --- | --- |
| Alkhajah et al. (27) | - | + | + | + | - | - | - | - |
| Chau et al. (28) | - | + | + | + | - | X | X | X |
| Gao et al. (29) | X | + | + | NI | NI | X | X | X |
| Gilson et al. (30) | X | - | + | NI | NI | - | - | X |
| Healy et al. (31) | - | + | + | NI | - | - | - | NI |
| Pronk et al. (32) | X | + | NI | NI | NI | X | X | X |

*+, low-risk of bias; -, moderate-risk of bias; X, serious-risk of bias; NI, no information provided*

*Table 3.3: Summary quality assessment of mixed methods studies (Mixed Methods Appraisal Tool)*

| Author (reference) | Qualitative | | | | Quantitative | | | | Mixed methods | | |
| --- | --- | --- | --- | --- | --- | --- | --- | --- | --- | --- | --- |
|  | Sources of data appropriate | Data analysis appropriate | Considered context | Researcher influence considered | Sampling appropriate | Sample representative | Measures appropriate | Response rate (≥60%) | Relevant design | Integration appropriate | Limitations considered |
| Grunseit et al. (33) | Y | Y | N | ? | ? | N | N | N | Y | Y | ? |

*Y, Yes; N, No; ?, can’t tell*

Overall score for Grunseit et al.: 0% (2/4 for qualitative component, 0/4 for quantitative component, 2/3 for mixed methods component)

*Table 3.4: Summary quality assessment of qualitative studies (CASP Tool for Qualitative Studies)*

| Author (reference) | Clear aim | Appropriate methods | Appropriate study design | Recruitment appropriate | Data collection appropriate | Researcher-participant relationship considered | Ethical issues considered | Data analysis rigorous | Clear statement of findings | Research valuable |
| --- | --- | --- | --- | --- | --- | --- | --- | --- | --- | --- |
| Chau et al. (7) | Y | Y | Y | Y | Y | ? | Y | Y | Y | Y |
| Dutta et al. (8)* | N | ? | NA | NA | NA | NA | NA | NA | NA | NA |
| Leavy et al. (9) | Y | Y | Y | Y | Y | ? | Y | Y | ? | Y |

*Y, Yes; N, No; ?, can’t tell; NA, not applicable*

**Dutta et al. failed the initial screening questions, so no further quality assessment was conducted, instead it was automatically deemed to have a high-risk of bias*

All RCTs were defined as having a high-risk of bias. This was primarily as a result of lack of blinding of participants which may have resulted in reporting or social desirability biases. However, due to the very nature of these types of health promotion interventions, is very difficult to mitigate this, although the use of objective measurement tools can help mitigate recall/reporting biases. Other prominent reasons for the high-risk of bias were as a result of the lack of researcher blinding to the allocated interventions and the use of subjective measurement tools resulting in recall or social desirability biases.

For the non-randomised trials, four were determined to be of serious-risk of bias (28–30,32), one of moderate-risk of bias (27), and one lacked enough information within one of the domains to allow a full assessment of risk of bias (31). The main reasons for giving an overall assessment of serious-risk of bias related to the lack of adequate control of confounders, the measurement of outcomes used (as a result of a lack of researcher blinding and the use of subjective measurement tools), and the selection of reported results (due to the presence of multiple outcome measurements within the outcome domain or multiple analyses of the intervention-outcome relationship).

For the mixed-methods study by Grunseit et al. (33), the qualitative component received a score of three-out-of-four and the mixed-methods component received a score of two-out-of-three. However, as the quantitative component received a score of zero, the overall score for paper was therefore also zero. The zero score for the quantitative component was due to the sample not being representative of the population under study, the lack of an objective measure of sitting time, and a poor response rate.

The CASP tool, used to determine quality of the qualitative studies, does not recommend the calculation of an overall score of quality, so a more narrative assessment of quality is required. The study by Dutta et al. (8) did not pass the screening questions as the aim of the research was not reported. Further, there were multiple quality issues highlighted with the paper including: a lack of use of direct quotes to support findings; the use of quantitative data to present qualitative findings; and an unclear purpose of conducting both focus groups and interviews which addressed the same issues and reported similar findings. Therefore, more formal assessment of quality was not undertaken, instead it was categorised as a low-quality study. The other two qualitative papers (7,9) passed the initial screening questions, but failed to explicitly state how the relationship between the researchers and the participants was considered. Furthermore, Leavy et al. (9) did not provide a clear statement of findings as there was little discussion of research that contradicted the findings, but supportive research was identified.

**References**

1. Higgins JPT, Altman DG, Gøtzsche PC, Jüni P, Moher D, Oxman AD, et al. The Cochrane Collaboration’s tool for assessing risk of bias in randomised trials. BMJ [Internet]. 2011 [cited 2017 Jun 26];343. Available from: http://www.bmj.com/content/343/bmj.d5928.long

2. Sterne JA, Hernán MA, Reeves BC, Savović J, Berkman ND, Viswanathan M, et al. ROBINS-I: a tool for assessing risk of bias in non-randomised studies of interventions. BMJ [Internet]. 2016 [cited 2017 Jun 26];355. Available from: http://www.bmj.com/content/355/bmj.i4919.full

3. Pluye P, Robert E, Cargo M, Bartlett G, O’Cathain A, Griffiths F, et al. Proposal: A mixed methods appraisal tool for systematic mixed studies reviews [Internet]. Montreal; 2011. Available from: http://mixedmethodsappraisaltoolpublic.pbworks.com

4. Critical Appraisal Skills Programme. CASP qualitative checklist [Internet]. 2017. Available from: http://www.casp-uk.net/checklists

5. Thomas J, Harden A. Methods for the thematic synthesis of qualitative research in systematic reviews. BMC Med Res Methodol [Internet]. 2008 [cited 2017 Jun 26];8(8). Available from: http://www.biomedcentral.com/1471-2288/8/45

6. Dixon-Woods M, Bonas S, Booth A, Jones DR, Miller T, Sutton AJ, et al. How can systematic reviews incorporate qualitative research? A critical perspective. Qual Res [Internet]. 2006 Feb 1 [cited 2017 Jun 26];6(1):27–44. Available from: http://qrj.sagepub.com/cgi/doi/10.1177/1468794106058867

7. Chau JY, Daley M, Srinivasan A, Dunn S, Bauman AE, van der Ploeg HP. Desk-based workers’ perspectives on using sit-stand workstations: a qualitative analysis of the Stand@Work study. BMC Public Health. 2014 Jul;14.

8. Dutta N, Walton T, Pereira MA. Experience of switching from a traditional sitting workstation to a sit-stand workstation in sedentary office workers. Work [Internet]. 2015 Aug 19 [cited 2015 Oct 23];52(1):83–9. Available from: http://content.iospress.com/articles/work/wor1971

9. Leavy J, Jancey J. Stand by me: qualitative insights into the ease of use of adjustable workstations. AIMS PUBLIC Heal. 2016;3(3):644–62.

10. Brakenridge CL, Fjeldsoe BS, Young DC, Winkler EAH, Dunstan DW, Straker LM, et al. Evaluating the effectiveness of organisational-level strategies with or without an activity tracker to reduce office workers’ sitting time: A cluster-randomised trial. Int J Behav Nutr Phys Act [Internet]. 2016;13. Available from: http://ovidsp.ovid.com/ovidweb.cgi?T=JS&PAGE=reference&D=psyc13&NEWS=N&AN=2016-54045-001

11. Chau JY, Daley M, Dunn S, Srinivasan A, Do A, Bauman AE, et al. The effectiveness of sit-stand workstations for changing office workers’ sitting time: results from the Stand@Work randomized controlled trial pilot. Int J Behav Nutr Phys Act [Internet]. 2014 Jan [cited 2015 Jan 23];11:127. Available from: http://www.pubmedcentral.nih.gov/articlerender.fcgi?artid=4194364&tool=pmcentrez&rendertype=abstract

12. Danquah IH, Kloster S, Holtermann A, Aadahl M, Bauman A, Ersboll AK, et al. Take a Stand!-a multi-component intervention aimed at reducing sitting time among office workers-a cluster randomized trial. Int J Epidemiol [Internet]. 2016; Available from: http://ovidsp.ovid.com/ovidweb.cgi?T=JS&PAGE=reference&D=medp&NEWS=N&AN=27094749

13. De Cocker K, De Bourdeaudhuij I, Cardon G, Vandelanotte C. The effectiveness of a web-based computer-tailored intervention on workplace sitting: a randomized controlled trial. J Med Internet Res [Internet]. 2016;18(5):e96. Available from: http://ovidsp.ovid.com/ovidweb.cgi?T=JS&PAGE=reference&D=prem&NEWS=N&AN=27245789

14. Donath L, Faude O, Schefer Y, Roth R, Zahner L. Repetitive daily point of choice prompts and occupational sit-stand transfers, concentration and neuromuscular performance in office workers: an RCT. Int J Environ Res Public Health. 2015 Apr;12(4):4340–53.

15. Dutta N, Koepp G, Stovitz S, Levine J, Pereira M. Using sit-stand workstations to decrease sedentary time in office workers: a randomized crossover trial. Int J Environ Res Public Health [Internet]. 2014 Jun 25 [cited 2015 Mar 17];11(7):6653–65. Available from: http://www.pubmedcentral.nih.gov/articlerender.fcgi?artid=4113835&tool=pmcentrez&rendertype=abstract

16. Evans RE, Fawole HO, Sheriff SA, Dall PM, Grant PM, Ryan CG. Point-of-choice prompts to reduce sitting time at work: a randomized trial. Am J Prev Med [Internet]. 2012 Sep [cited 2015 May 19];43(3):293–7. Available from: http://www.ncbi.nlm.nih.gov/pubmed/22898122

17. Graves LE, Murphy RC, Shepherd SO, Cabot J, Hopkins ND. Evaluation of sit-stand workstations in an office setting: a randomised controlled trial. BMC Public Health [Internet]. 2015 Jan 19 [cited 2015 Nov 23];15(1):1145. Available from: http://bmcpublichealth.biomedcentral.com/articles/10.1186/s12889-015-2469-8

18. Healy GN, Eakin EG, Owen N, Lamontagne AD, Moodie M, Winkler EAH, et al. A cluster randomized controlled trial to reduce office workers’ sitting time: effect on activity outcomes. Med Sci Sports Exerc [Internet]. 2016;48(9):1787–97. Available from: http://ovidsp.ovid.com/ovidweb.cgi?T=JS&PAGE=reference&D=prem&NEWS=N&AN=27526175

19. Neuhaus M, Healy G, Dunstan D. Workplace sitting and height-adjustable workstations: a randomized controlled trial. Am J … [Internet]. 2014 [cited 2015 Mar 19]; Available from: http://www.sciencedirect.com/science/article/pii/S0749379713005369

20. Parry S, Straker L, Gilson ND, Smith AJ. Participatory workplace interventions can reduce sedentary time for office workers: a randomised controlled trial. PLoS One [Internet]. 2013 Jan [cited 2015 Feb 10];8(11):e78957. Available from: http://www.pubmedcentral.nih.gov/articlerender.fcgi?artid=3827087&tool=pmcentrez&rendertype=abstract

21. Puig-Ribera A, Bort-Roig J, Gonzalez-Suarez AM, Martinez-Lemos I, Gine-Garriga M, Fortuno J, et al. Patterns of impact resulting from a `sit less, move more’ web-based program in sedentary office employees. PLoS One. 2015 Apr;10(4).

22. Swartz AM, Rote AE, Welch WA, Maeda H, Hart TL, Cho YI, et al. Prompts to disrupt sitting time and increase physical activity at work, 2011-2012. Prev Chronic Dis [Internet]. 2014 Jan [cited 2015 May 19];11:E73. Available from: http://www.pubmedcentral.nih.gov/articlerender.fcgi?artid=4008949&tool=pmcentrez&rendertype=abstract

23. Urda JL, Lynn JS, Gorman A, Larouere B. Effects of a minimal workplace intervention to reduce sedentary behaviors and improve perceived wellness in middle-aged women office workers. J Phys Act Health [Internet]. 2016;13(8):838–44. Available from: http://ovidsp.ovid.com/ovidweb.cgi?T=JS&PAGE=reference&D=prem&NEWS=N&AN=26998705

24. Priebe CS, Spink KS. Less sitting and more moving in the office: Using descriptive norm messages to decrease sedentary behavior and increase light physical activity at work. Psychol Sport Exerc [Internet]. 2015 Jul [cited 2015 Apr 3];19:76–84. Available from: http://www.sciencedirect.com/science/article/pii/S1469029215000230

25. Gordon A. A theory-based pilot study to decrease sitting time in the workplace - Dissertation Thesis. Arizona State University, Arizona; 2013.

26. Tobin R, Leavy J, Jancey J. Uprising: An examination of sit-stand workstations, mental health and work ability in sedentary office workers, in Western Australia. Work [Internet]. 2016;55(2):359–71. Available from: http://ovidsp.ovid.com/ovidweb.cgi?T=JS&PAGE=reference&D=prem&NEWS=N&AN=27689593

27. Alkhajah TA, Reeves MM, Eakin EG, Winkler EAH, Owen N, Healy GN. Sit-stand workstations: a pilot intervention to reduce office sitting time. Am J Prev Med [Internet]. 2012 Sep [cited 2015 Apr 30];43(3):298–303. Available from: http://www.ncbi.nlm.nih.gov/pubmed/22898123

28. Chau JY, Sukala W, Fedel K, Do A, Engelen L, Kingham M, et al. More standing and just as productive: effects of a sit-stand desk intervention on call center workers’ sitting, standing, and productivity at work in the Opt to Stand pilot study. Prev Med reports [Internet]. 2016;3:68–74. Available from: http://ovidsp.ovid.com/ovidweb.cgi?T=JS&PAGE=reference&D=prem&NEWS=N&AN=26844191

29. Gao Y, Nevala N, Cronin NJ, Finni T. Effects of environmental intervention on sedentary time, musculoskeletal comfort and work ability in office workers. Eur J Sport Sci [Internet]. 2016;16(6):747–54. Available from: http://ovidsp.ovid.com/ovidweb.cgi?T=JS&PAGE=reference&D=prem&NEWS=N&AN=26529590

30. Gilson ND, Ng N, Pavey TG, Ryde GC, Straker L, Brown WJ. Project Energise: Using participatory approaches and real time computer prompts to reduce occupational sitting and increase work time physical activity in office workers. J Sci Med Sport [Internet]. 2016;19(11):926–30. Available from: http://ovidsp.ovid.com/ovidweb.cgi?T=JS&PAGE=reference&D=prem&NEWS=N&AN=26922132

31. Healy GN, Eakin EG, Lamontagne AD, Owen N, Winkler EAH, Wiesner G, et al. Reducing sitting time in office workers: short-term efficacy of a multicomponent intervention. Prev Med (Baltim) [Internet]. 2013 Jul [cited 2015 Apr 30];57(1):43–8. Available from: http://www.ncbi.nlm.nih.gov/pubmed/23597658

32. Pronk NP, Katz AS, Lowry M, Payfer JR. Reducing occupational sitting time and improving worker health: the Take-a-Stand Project, 2011. Prev Chronic Dis [Internet]. 2012 Jan [cited 2015 Jul 1];9:E154. Available from: http://www.pubmedcentral.nih.gov/articlerender.fcgi?artid=3477898&tool=pmcentrez&rendertype=abstract

33. Grunseit AC, Chau JY-Y, van der Ploeg HP, Bauman A. “Thinking on your feet”: a qualitative evaluation of sit-stand desks in an Australian workplace. BMC Public Health. 2013 Apr;13:365.
