# Supplementary material for: The development, implementation and evaluation of interventions to reduce workplace sitting: a qualitative systematic review and evidence-based operational framework
Source: BMC Public Health. 2018 Jul 4;18:833. doi: 10.1186/s12889-018-5768-z (PMC6033205; doi:10.1186/s12889-018-5768-z)
Supplement: Supplementary file 4 — Raw Qualitative Data. (DOCX 46 kb) [file 12889_2018_5768_MOESM4_ESM.docx]

**Additional File 4: Raw Qualitative Data**

**Brackenridge 2016 RCT:**

Emails and information booklet were delivered as planned. Further intervention fidelity data were not collected.

Not great usage of LUMOback activity tracker - issues with using technology, reactions to it. No one at 12-months was using it.

From discussion section:

- Authors stated that the modest changes in sitting time (when compared to using sit-stand desks) may be explained by "for occupations that are primarily desk-based, the work environment may limit capacity for change, and office equipment and task design modifications are needed to enable greater changes in sitting and standing during the work day".

- “The organisation also has a key focus on employee health and was likely already receptive to workplace health improvements. Additional initial strategies focusing on knowledge and cultural change may be needed in workplaces without a champion or health focus.”

Stated that this shows impact of organisational support and activity tracker strategies on office workers' sitting time.

*No theory used/documented.*

*No co-production used - although use of workplace champion.*

*Multi-component intervention - although stated that it was an organisational-level intervention, but the availability of Trackers with individual feedback provides some individual-level input.*

*Some tailoring - via workplace champion.*

*No cost-effectiveness reported - although suggested that it was a low-resource intensive intervention.*

*No qual data to present what worked and why and determine what the impact of organisational support was.*

**Brackenridge 2016 Protocol: linked to study above**

No theory reported (although some mention of SEM, but not highlighted that this was used in the development of the intervention)

**Chau 2014 RCT:**

Strength of study (in discussion) - "having strong staff and management support for evaluation".

From discussion "Upfront costs is a likely barrier for many workplaces to roll out this type of intervention on a larger scale, so demonstrating longer-term effectiveness is important. Evaluating other lower cost behavioural strategies aimed at reducing extended sitting at work is also warranted, to determine whether similar reductions in sitting time can be achieved."

*No theory used/documented*

*No co-production used*

*Single-level intervention*

*No tailoring*

*No cost-effectiveness reported*

**Chau 2014 Qual: linked to study above**

Motivations grouped into 3 themes:

- Curiosity to try something new - "...to give it a test without actually having to commit to it really, long term… the trial aspect was very interesting."

- Interest in potential health benefits - "I wanted to know that I wasn’t putting strain on my cardiovascular system and arteries by sitting 8 hours at a time and I just wanted to see if it had a difference to my energy levels and my problems with my back."

- Relevance to the participant's own and organisation's work - For these participants, being able to contribute to research and workplace policy, as well as experiencing first-hand what it was like to use a sit-stand workstation were motivators for engaging in the trial (quote from paper not from participants).

- Small number of participants cited extrinsic factors for trialling workstation e.g. encouragement from colleagues or managers.

General impressions of using sit-stand desks grouped into 3 themes:

- Surprise and delight - used device more than thought and unexpected benefits such as improved posture and increased alertness (no participant quote).

- Impact on ability to work - negative impressions impacted on comfort and work ability due to specific design issues of device (no participant quote).

- Having choice - "I just enjoyed the choice, you know, since it’s been gone, you know, I sit there and go “I should stand up and do this task”. Yeah purely for choice and, yep, being able to make that active choice."

Patterns of use grouped into 3 themes:

- Task-based routine - "If I need to get stuck into writing something or I need to go through to one of my files, I’ll sit back down again, and then if I’m replying to emails, or you know just working on one document on the screen, then I’ll get back up again."

- Time-based routine - "When I saw the clock tick over the hour, I was like, “get up”."; "I’d tried to do half hour on and half hour off."; "Probably for around about 30 – 40 minute bouts, I suppose, during the day. Probably once every two hours or so."; "I got in and normally tried to stand straight away because I felt good standing up and it was probably best to stand first thing in the morning."; "It was better concentrating in the afternoon because you tend to get a bit dozy after lunch, so I think standing after lunch was much better."; "It’s a good change for me to be able to stand, and definitely the afternoon is a point where I have a lag. It’s a dead hour after lunch. It’s always hard getting over that hump into the afternoon."

- No particular routine - "I didn’t stand for long periods. I stood and sat - I was like a Jack-in-the-box. I was up and down, up and down, rather than standing for a long time and sitting for a while."; "I didn’t really assign a pattern to it, I was just like, “oh, I’ve been sitting for a bit, I’ll stand up or I’m getting a bit tired”."

- Some participants described standing to work as similar to developing a new habit and some felt they were able to stand longer - personal challenge - "I used to look at the clock and go “I’ll just do 20 minutes more” and, like, it was always like a little self-competition going."; "I think they say it takes 3 or 6 weeks to develop a new habit and this is really kind of like having a new habit."

Barriers to using sit-stand desks in a standing position grouped into 2 themes:

- Working in the open plan office - "I did feel if I was doing something confidential that it was more on show, so I was a little bit wary of that."; "When I’m on the phone standing up I feel a little bit conscious because I feel like I’m shouting out across everyone and I’m sort of distracting people next to me."; "I think if everybody was standing/sitting all the time, nobody would actually care, people would get used to it because if I had people moving all the time and I was standing up, I would probably learn to concentrate more with those distractions."

- Sit-stand workstation design - "I think it kind of depends on your working style, I think if you are somebody who just does nothing with a pen and paper or rarely or very rarely does, it’s perfect because you know you’ve got everything there that you need, but I, I’m a person who often needs to stop typing and starting jotting things down or mapping things out or, you know, with a good old fashion pen and paper, and then you’re like “okay, oh that’s wobbling”."; "I found it cut down on my desk space which was a little bit annoying sometimes."

- Some mentioned physical discomfort - "Sometimes I would be typing as I was standing and going “oh wow I’m getting major pain … in my forearms” and so that was “oh it would be nice if it was already set at your height, perfectly” so you didn’t have to (adjust it every time)."

Facilitators grouped into 3 themes:

- Supportive work environment conducive to standing - "It does make it a lot more sociable environment… I think it encourages the interaction with going to speak to people as opposed to just always reverting to an email and sitting in your own little silo."; "You don’t feel alone, you know, you’re going to stick out like a sore thumb when you are doing work but it’s good when other people do."

- Perceived physical health benefits - "I found my back hurt less as well, cause I’ve got back issues and I found it a lot better, and I was worried about it, you know, maybe it will be weird, but my back has hurt a lot less standing up than sitting."; "I felt better at the end of the day. I felt a bit more not as tired at the end of the day."

- Perceived work benefits - "I was less fidgety I found it really good, cause I could jump up and down and I found I got a lot more done."

Willingness to continue with sit-stand desks - mixed responses

- Negative: "I don’t want to stand up, but not so much that I would knock it back… I would get used to it and I would build it in to my day but I wouldn’t go looking for it."; "Honestly, no and that’s not because I didn’t like standing up as much… it was the desk… I found it quite a challenge with just the way I work."

- Positive: "I would love to have it back, I just think it’s great to have that option of sitting standing and not have to be tied to sitting down all day."; "I would actually like one that can be adjusted to be, you know, your height and also can be adjusted as to how far it is from your eyes."

- Alternatives: computer prompts to stand up or having access to a standing ‘hot desk’.

Changes in sitting and standing behaviour since the trial - general comments re. increased awareness

- "I just learnt to tolerate to stand a little longer now. So in a lot of, like, meetings I go to I get up out of my seat and stand."

- "When I’m at home on my laptop, I take it down to the kitchen bench because it’s high enough for me to stand there and do what I need to do, so I really prefer standing now."

- "Standing on trains… 70% of my choice and 30% is simply because there are no seats… I’ve sat all day at work so I just would stand but yeah, now I think I like standing more."

- Change in perspective - "There’s definitely more (standing). I think it’s the awareness of all this happening and I think people think “I can stand for a meeting. It’s okay to stand”… I was actually in an external meeting yesterday with my colleague and she got up and stood up and the facilitator kind of looked at her as if to say “why are you standing up?” … I just thought, you know, it’s quite common in our workplace to do that now, but clearly this woman wasn’t used to that sort of thing happening."

Further qualitative info from discussion section - quoted from authors:

"The sit-stand workstations were implemented within the study workplace through a collaborative approach. Managers responsible for initiating the trial promoted the study as an opportunity to evaluate how effective and acceptable sit-stand workstations were as a sitting reduction strategy, before committing to a larger roll out, given the lack of evidence to guide decision making. Employees felt they were contributing to both research and organisational health policy, as they were consulted about their views on the workstations while they were given an opportunity to participate in a new area of health promotion that aligned with the organisations purpose. This is reflected in their reasons for participating, which encompassed both individual motives (e.g., trying something new, interest in potential health benefits, flexibility to work standing up) and organisational factors (e.g., relevance to their own and their organisation’s work around workplace wellness and cardiovascular health)."

"The collaborative engagement of employees in the Stand@Work study is consistent with previous qualitative findings from office workers, that implementing any workplace sitting reduction strategy is both the responsibility of individual employees and organisational management, and evidence that greater investment in educating and motivating workers to use sit-stand workstations results in greater uptake. The collaborative approach taken in this study is likely to have contributed to participants’ willingness to try using the sit-stand desk in a standing position. This is consistent with one study where the intervention group comprised sedentary behaviour researchers who were most likely highly supportive of working in a standing posture, educated on the potential health effects of prolonged sitting, and applying ‘sit-less’ strategies in the workplace already with management support."

"Occupational health and safety practitioners have emphasised the importance for workers to have choice over whether they sit or stand to work, and expressed concerns regarding the potential for perceived coercion when implementing any sitting reduction strategy as employees could feel pressured to stand for extended periods, highlighting a tension between optional standing versus compulsory standing. Stand@Work overcame this with clear instructions from the start that participants did not have to stand to work, and that their participation was an evaluation to inform their workplace wellness program and future procurement decisions."

"From an employer perspective, one of the potential advantages of this type of environmental modification to reduce sitting is that work time is not interrupted. Usual tasks can still be undertaken in the same location, and from our participant’s feedback, there may also be productivity improvements due to increased alertness, concentration and reduced fatigue, especially later in the day."

"It is too early to make a strong business case for large scale investment in sit-stand workstations, as the health and productivity benefits are yet to be quantified and there is a need for more evidence about longer term use and maintenance of reductions in sitting time reported elsewhere. It would also be important to explore lower cost options for reducing sitting because not all workplaces may be able to install or afford sit-stand workstations."

"Participants also noted the emergence of a ‘sitting less culture’ within the workplace and there are a number of ways this could be encouraged. A menu of sitting reduction options could be designed and provided to workplaces to allow more choice for employers and employees."

*Although this is the same study as above, this talks about the fact that a collaborative approach was used.*

**Chau 2016 Non-Randomised:**

From discussion: "Organisations considering sit-stand desks as part of their workplace wellness practice would need to balance the costs associated with purchasing new furniture with that of potential productivity trade-offs."

From discussion: "A major strength of this study was the strong engagement with upper level management in the partner organisation at all stages of study planning, implementation, and evaluation. This reflected the company's image as being forward-thinking, open to change, optimistic, and person-centric. Clear management support facilitated the logistics of conducting this study: the procurement department liaised with the corporate furniture provider, Zenith, who agreed to provide and install sit-stand desks for one team as a free trial; the risk management department monitored the planning and implementation process; the communications department wrote about the trial in the staff newsletter; and the health and wellness manager coordinated all parties and arranged for the research team to access in-house productivity measures. At the team level, the team leader of the intervention group was proactive in the study's development phase and attended planning meetings prior to trial commencement. The control team was led by two leaders who worked part-time and were relatively less engaged and communicated less frequently with the research team. In terms of evidence-based practice, this was a valuable project for the partner organisation because the relevance of the setting and resulting data meant a greater level of buy-in from the business. The results have informed an overall strategy to address sedentary work practices across the organisation, one that looks at both work environment and behaviour change. Evidence-based strategies are increasingly in demand in Australian businesses to help inform well-being programmes, to allocate resources, and to determine return on investment. This partnership between researchers and practitioners has provided an effective model for future cooperation in workplace health promotion."

*No theory used/documented*

*No co-production used - although comments on management involvement in discussion section (see qual comments)*

*Multi-component intervention - sit-stand desks, email prompts*

*No tailoring*

*No cost-effectiveness reported - mention of cost-effectiveness in qual comments.*

**Danquah 2016:**

Fidelity of the intervention reviewed in terms of “dose” delivered, “dose” received and harm

Dose delivered

- all 5 intervention components were implemented following similar procedures at all 4 workplaces. All lectures and workshops had been held according to a similar schedule at all sites, and all offices set goals during the workshops.

- all sites had provided facilities for standing and walking meetings; at 2 workplaces, new tables had been acquired, at the other 2 sites tables had been relocated.

- emails and text messages had been sent out as planned

- all planned meetings with managers and ambassadors had been held before the start of the intervention and at 1 month.

Dose received

- 86% participants felt that management had supported the project

- 81% felt a sense of community in relation to the project

- 79% knew where to have standing meetings and 85% of these found the facilities adequate

- 89% knew where to have walking meetings and 92% of these found the facilities adequate

- in intervention group, 76% participated in the workshops

- 83% had set goals, 73% signed up for emails, 42% signed up for text messages

Harm

- 8% experienced an increase in the level of noise

- <6% experienced persistent pain in back/lower back or legs/feet, decreased personal productivity, decreased meeting quality, or other negative consequences

Intervention developed using intervention mapping. When the behavioural and environmental outcomes were ascertained, specific determinants affecting these outcomes were addressed. Specific activities were identified for each determinant using social cognitive theory, Rogers' diffusion on innovations theory and goal-setting theory.

Pilot study conducted in a test office, contributed to the development of the intervention. As part of the formative evaluation, observations were carried out during one day at the test office, focusing on workplace facilities, routines, interactions between employees and meeting frequencies and facilities. A focus group interview with 5 volunteers from the test office was conducted to discuss and develop ideas for intervention components and implementation.

After initial development, the intervention was trialled at the test office with 15 participants. After 1 month, the intervention was evaluated qualitatively with participants and minor adjustments made to the intervention.

From discussion:

- Multicomponent intervention including organizational, environmental and individual components.

- Context was participants who were accustomed to a sit-stand desk, but before the intervention, the majority did not use them.

- Even though the individual level was less extensive in this intervention (compared to others which used face-to-face consultations and follow-up by telephone), reductions in sitting time were comparable and therefore this intervention was cheaper and easier to disseminate on a larger scale.

- Large diverse population from a variety of workplaces

- Participants had different types of office work and came 20 from both public and private-sector workplaces. In addition, the 19 offices represented different office environments with small as well as large offices, customer/citizen interaction, various meeting frequencies and gender composition.

**De Cocker 2015 Intervention Development:**

*Discussion mentioned focus groups but no mention of these previously...*

From discussion:

- "full-time employees were less likely to request the advice compared to part-time workers, so it may be that ‘time’ was an issue. Focus group interviews among employees and managers also revealed that productivity concerns and loss of time were barriers for the implementation of intervention strategies to reduce or interrupt sitting at work."

- "Discussing the advice with others could also overcome barriers, such as the awkwardness of standing or the fear of disturbing others while standing, which were reported by employees and executives in focus group interviews".

- Findings from this study "suggest that interrupting sitting is more achievable to implement than replacing longer periods of sitting by standing at work."

Intervention based on previously developed computer-tailored PA interventions.

Tailoring constructs were based on Theory of Planned Behaviour (TPB) with the concept of goal-setting integrated (goal-setting and action plans operate within Self-Regulation Theory). Also concepts of Self-Determination Theory were accounted for (users were allowed to choose which sections they wanted to focus on).

**De Cocker 2015 RCT: (linked to study above)**

From discussion: quoted as a limitation "we recruited participants in only 2 companies, with worksites in 3 different settings, probably resulting in different workplace cultures" - "may compromise generalisability"

Self-reported measures showed positive changes but objective measures did not corroborate this.

*See info above re. intervention development*

**Donath 2015:**

From discussion:

"The observation that people respond differently to the appearance of point of choice prompts is in line with previous studies on individual patterns of how prompts appeal to the participants [37,38]. These studies pointed to personal (e.g., socio-economical, volitional, motivation, educational) and environmental factors (job profile, applicability, reasoning of the prompt target) that can affect the compliance. In this regard, stressful jobs [39] and the presence of health impairments [40] are negatively associated with the use of HAWDs [sit-stand desks]. Thus, it seems reasonable to assume that the application of point of choice prompts [41] should reflect individuals’ needs, backgrounds and the specific working environments [42,43] that, as a consequence, might enable a more successful occupational health promotion in terms of sit-to stand transfers."

"Health promoting worksite programs should be embedded in policy strategies that aim at tackling unhealthy behavior in general."

*No theory used/documented.*

*No co-production used.*

*Simple intervention - sit-stand desks (as standard to intervention and control group) with prompts.*

*No reported tailoring.*

*No cost-effectiveness reported - although stated in conclusions that prompts are a "cost-saving instrument".*

**Dutta 2014 RCT:**

From results: "Most participants reported experiencing increased fatigue, especially in the lower back and lower extremities, during the beginning of the intervention as they were adjusting to the SSDs and increased standing time at work. For all participants, the back fatigue/discomfort was no longer present by the second week of the intervention. The major complaint regarding the SSDs from usability perspective was the loss of work-surface compared to traditional sitting desk. Despite that, 26 out of the 28 participants at the end of the study reported willingness to continue using SSDs beyond the end of the study, thus had the desks permanently installed."

From discussion:

"Participants enjoyed the flexibility to be able to sit or stand while working which was reflected by the overwhelming majority choosing to permanently keep the SSDs".

"The SSDs used for this study are in the $400 to $900 range, which is in the price range for high quality office chairs, and affordable relative to high quality office furniture that would include the desk and the chair."

"This study was done in the ‘real-world’, at the worksite and the natural workflow of the employees did not appear to be disrupted to any significant negative extent."

*No theory used/documented.*

*No co-production used.*

*Single-level intervention - sit-stand desks*

*No reported tailoring.*

*No cost-effectiveness reported*

**Dutta 2015 Qual: (linked to study above)**

Individual interviews:

96% of participants reported having a positive experience using sit-stand desks and they indicated that they wanted to continue using the desks.

Reported benefits of sit-stand desks included: increased levels of energy, focus, alertness (74%); increase in social energy ("buzz") in the workplace (59%) due to increased communication; alleviation of back pain (19%).

Reduction in desk space was a frequent complaint associated with sit-stand desks use (25%) and loss of privacy (11%).

37% reported some MSK soreness for first 2 weeks of sit-stand desk use, but this discomfort gradually dissipated.

Focus groups:

Physical environment changes:

- limited availability of desk surface was major drawback of sit-stand desks

- anti-fatigue mats were found to be useful

Health changes:

- short-term MSK discomfort during an early adjustment period, but disappeared after 2 weeks

- some found alleviation of low back pain with sit-stand desks

- sit-stand desks led to heightened awareness of posture, ergonomics and frequent postural adjustments which induced them to move about more frequently.

Social environmental changes:

- standing facilitated workplace interactions, specifically face-to-face communications

- noise level was not disruptive, no major privacy concern once workers had adjusted to the use of sit-stand desks

Productivity changes:

- participants noted an increase in energy and focus, but worker productivity did not change with the use of sit-stand desks.

From discussion:

- most participants noted that the benefits of standing outweighed the drawbacks associated with space limitations

- "Non-participants suggested that the presence of standing co-workers may have had a positive impact on workplace productivity. By being able to view standing co-workers, some individuals suggested that they were provided with cognitive cues to interact with others on work-related matters. For example, seeing a particular co-worker standing might remind an individual of a project step that needed to be reviewed."

- "As had been the case with other sit-stand desk interventions, participants did not perceive a decrease in productivity."

**Gao 2016:**

From discussion: "Collectively, these findings suggest that implementation of sit–stand workstations alone is not sufficient, and highlight the need for effective guidance (i.e. tailored counselling) to use the sit–stand function for reducing sedentary time, including adequate instruction and promotion of the potential health benefits.”

*No theory used/documented.*

*No co-production used.*

*Single-level intervention - sit-stand desks*

*No reported tailoring.*

*No cost-effectiveness reported*

**Gilson 2016:**

Workshop identified a menu of 20 "sit less, move more" strategies, themed into 4 occupational contexts of desk-tasks, meetings, work breaks and travel.

From table 2:

Theme 1: Standing and moving during desk tasks

Theme 2: Standing and moving in or between meetings

Theme 3: Standing and moving during work breaks

Theme 4: Active travel to and from work

From discussion:

"More recently, studies that have focused on sitting and chronic disease in office workers have advocated and used participatory approaches as a means of promoting ownership of occupational sitting reduction strategies and facilitating commitment to sedentary behaviour change."

"A main finding of our study is that participants identified a more comprehensive ‘menu’ of 20 strategies [compared to previous studies], themed into four specific occupational contexts. This ‘menu’ is valuable for employers and practitioners interested in providing office workers with a range of choices and opportunities for reducing and interrupting sitting in different situations, and with occupational groups who have different job demands and daily routines. Ongoing testing now needs to occur to assess if the ‘menu’ is exhaustive and replicable with other office-based samples."

"Our data adds to the view that a participatory approach allied with real time prompts may be valuable. However, it is important to consider that multi-level interventions, that target the individual, environmental, organisational and policy domains of the office work system are more likely to have a comprehensive and sustainable impact on occupational sitting, than interventions that target any one domain in isolation."

*Managers of each team distributed the recruitment emails - perception of management support*

*Participatory intervention design*

*Some individual-level tailoring for the prompts to fit in with work routine*

*No cost-effectiveness reported*

**Gilson 2012:**

From discussion:

"the ‘hot’ desk concept fits well with employer financial constraints and the cost concerns of allocating height-adjustable desks to individuals"

*No theory used/documented.*

*No co-production used.*

*Multi-component intervention - sit-stand desks + education*

*No reported tailoring.*

*No cost-effectiveness reported*

**Gorman 2013 publication:**

From discussion:

"It is important to note that participants were not provided with information or education regarding the benefits of reducing prolonged workplace sitting and/or how the new building could help them achieve this. Thus, the change observed could be considered to be primarily reflective of these large-scale environmental changes. Effects may be more substantial if environmental change occurs in combination with interventions based on individual (e.g., goal setting), social (e.g., addressing cultural norms around sitting and moving at the workplace), and organizational-level (e.g., visible organizational support; policies to support standing and moving) change strategies."

"Interestingly, given that the new building was designed to be ‘activity-permissive’, there were minimal changes in stepping time pre-post move." - ?focus on activity rather than sitting less per se.

"Although large-scale modifications to the physical environment are expensive, once built, the intervention (i.e. the structural changes of the building) is on going, and any potential benefits impact all employees (i.e., not just research participants). Furthermore, this study suggests that such benefits may not just be limited to change in activity, but also to work-related outcomes."

*No theory used/documented.*

*No co-production used.*

*Single-level intervention - environmental-level change only*

*No reported tailoring.*

*No cost-effectiveness reported*

*However, "intervention" was a planned change - a natural experiment rather than a formal experimental process*

**Gorman 2012 dissertation: (linked to above paper)**

From results:

"Participants reported that they spent less time sitting at the new workplace [83.8% (7.0) vs. 79.4 % (13.3)]. They also reported that people at their workplace generally spent less time sitting at follow-up [87.1% (4.7) vs. 82.5% (8.0)]. However, they did not change their opinion on how much time they would like to spend sitting at their workplace. At both time points participants preferred to spend approximately 60% of their workplace time sitting. Their perceptions of their control of workplace standing changed; at baseline most did not think it was up to them whether they sat or stood at work and at the final assessment they neither disagreed nor agreed with this statement. Lastly, at baseline and follow-up participants agreed that if they have to sit a lot at the workplace, they “feel like exercising in my own time”."

"Participants generally enjoyed the new workplace [median 5 (IQR = 4 to 5)] and did not want to return to the original workplace [median 1 (IQR = 1 to 2)]. They also agreed that the new workplace had improved their productivity [median 4 (IQR = 3 to 5)]. They were neutral regarding if the workplace had decreased their time sitting at the workplace [median 3 (IQR = 2 to 4)] or outside of the workplace [median 3 (IQR = 2 to 3)] and did not think that their standing while outside the workplace had decreased [median 2 (IQR = 2 to 3)] with the move to the new workplace."

**Graves 2015:**

Patterns of workstation use:

- Variation when participants self-selected standing: "I did start off the first week of using it [the sit-stand workstation] almost all of the day"; "I did try and use it [the sit-stand workstation] at first even like every half an hour or so, or like quite often and then if I sat down I would try and use it again in half an hour or so and then it got to, oh I’ll do 5 or 10 min every hour"; this then tailed off over time "I think towards the end I tend to sit down a lot … I think it [use of the sit-stand workstation] just tailed off in the end".

Factors that have potential to influence workstation use:

- Workstation design, social environment, habits, and alertness

- Interplay between workstation design an type of task that could be completed in a standing position e.g. "Depends what work you had on because we use files a lot and it [the sit-stand workstation] wasn’t you know very good for with files and things were you tend to sit down".

- Non-sturdy nature of design

- The social environment had both a positive and negative impact upon workstation use. Some participants noted trepidation and feeling self-conscious in an environment where colleagues were working in seated positions. In addition there was a degree of consideration of others in the environment as a result of them standing whilst others were sitting. "Initially we were like uh God I’m standing up everyone else is sitting down …….. some people just felt a bit self-conscious erm just because they were standing up and everyone else around them wasn’t maybe that made them feel uncomfortable"; "If people were coming in to see these people (colleagues in close proximity) I sat down not to be a distraction so they can concentrate on what they’re doing".

- Support from peers "You would see someone else pop up and use theirs so you would think, oh yeah I’ll use mine"

- Once novelty had worn off, participant forgot to work in standing position "After a couple of weeks you started to decline, so there was almost like a novelty effect and then people were too busy to think about standing so they just reverted to sitting."

- Perceived concentration and alertness, some felt "I don’t know whether standing up and being able to see everyone more was a bit of a distraction as well but yeah I did find I couldn’t concentrate as much [when standing] and I’d need I think it’s just a natural thing to sit down and have all your things around you"; whilst others felt "I think it [standing] kind of makes me more productive straight away."

From discussion:

"The social environment and in particular the presence and actions of others were reported to support and deter standing work. For example, seeing co-workers standing prompted a transition from seated to standing work, while other workers cited feelings of self-consciousness as a reason to stay seated. Future trials could examine the effect of additional intervention strategies (e.g. workplace champions, wider workforce education on the intervention and its aims) to the simple provision of a standing workstation. Such strategies may additionally support sustained use of the workstation, and prevent workers from reverting back to the habit of seated working, which was observed in the present trial."

*Managers of 11 departments were initially approached for consent to participate prior to individual-level consent being sought - management involvement*

*Cost of sit-stand workstation: £360-375.*

*Single-level intervention*

*No theory used/documented.*

*No co-production used.*

*No reported tailoring.*

*No cost-effectiveness reported*

**Grunseit 2013:**

3 group interviews, 4 people in each

Initiation of use:

- no systematic or formal prompt

- the idea of using standing option was lost in range of other changes and tasks that accompanied the refurb

- reasons for first use - 1) anticipated health benefit or 2) experimentation with no expectation or because of external prompting

- health-driven initiative:

"I think I must have had a back ache at the time or something and I thought it sounded like a really great idea to be able to spend some time in the day standing rather than sitting the whole day."

Those who reported to be motivated for health reasons, were also "early adopters" and continued to be committed and enthusiastic users. Thus the potential health effects of providing an option for standing were a strong motivator for some employees.

- experimentation or external prompting:

For some it was more of a trial or prompted directly or indirectly by others "I wasn’t using it, but I’ve got a bit of a bad back and bit of a shoulder injury as well. So, for some reason it just never occurred to me to actually wind it up and a colleague said to me you should give it a go, so I did."

If no specific motivation, use can be left to happenstance: "So in the first instance it’s a personal individual thing but if someone becomes enthusiastic about it and receives the benefits then it’s quite likely that that will impact other people in the workplace. Conversely, if no one in the workplace is trying it, it takes an individual to stand up to try it and if everyone else is not then that may also impact."

Maintenance of use:

- health/physical impacts

Positive physical "Today I’ve got it up and I’ve a bad back anyway so it’s good you know to actually, when you stand it gets a bit easier."

Energy "I still have the same level of activeness, if not probably more. Actually I still feel pretty energised when I get home."

Standing and also moving more "And I do tend to move around my office a lot more as well. So I will go over and get something or I’ll walk out where as if you have to get up and walk away from your chair. I’m probably less likely to do it I’ll save it as a group of things so I’ll only have to go down once."

Negative physical "I can move sometimes with difficulty but standing in one spot is putting more pressure on my back, and automatically start after a while it just shoots pain down the legs. . . There is nothing wrong with the desk, it’s me."

- perceived work productivity / mental impacts

Positive "I think you do associate sitting with relaxing where as when I’m standing I am definitely more alert and far more productive."

Negative "Maybe you for sitting, maybe that impacts whether or not you use the desk or not, ‘cause I really can’t, I don’t feel focused at all when I’m standing. I’m shuffling from foot to foot and I’m usually just reading a document but the screen is too close or too it’s far away or my arms are too funny or whatever it might be. It’s just not comfortable. . .I don’t feel in the zone as it were."

Tasks being performed dictated position of desk but this was varied from person to person. Instead, some linked their preferences and productivity whilst sitting or standing to their habitual styles and past modes of working e.g. feeling more productive standing because ex-military where you were always making decisions on your feet.

- desk/office set-up/context

Practicalities of how set-up - cables, space, lack of storage space, wider office environment - changing things to standing height

[Effect of manual vs electric desk (not relevant here, some staff had manual sit-stand desks, some had electric, some had both - a sub-theme identified)]

From discussion:

"One group committed to using the standing option prior to installation and were persistent and frequent users. Any barriers encountered were resolved, work-arounds instituted to enable continued use of the desks. A second group also reported enjoying using the standing option but the discovery was more unexpected. Like those who were prior committed users, these newly committed users continued to stand regularly, even enthusiastically, but unlike the former were not necessarily convinced of the idea before trying the desks. Commitment arose out of perceived improved productivity and/ or experience of a health benefit (either resolution of a health problem or increased energy) and/or a good fit with established work habits after experimentation. A third, “uncommitted” group also experimented with using the desks in standing mode, but further use was undermined by difficulties encountered when transitioning or feeling ill at ease standing whilst working. Second-hand reports suggested a fourth group who were not interested in even trying the desk in the standing mode."

*Single-level intervention - opportunistic, part of previously planned refurb*

*No theory used/documented.*

*No co-production used.*

*No reported tailoring.*

*No cost-effectiveness reported - although, this was stated in conclusion "The one-off cost but potential continuing benefit of the desks makes them a viable option for workplaces looking to provide employees with options to reduce sitting at work."*

**Healy 2013:**

From discussion:

"A message to get up at least every 30 min is widely advocated within the ergonomic and occupational health and safety disciplines. The significant increase in sit–stand transitions and approximately 50% reduction in prolonged sitting time suggest

such regular postural changes may be feasible among office workers performing administrative tasks."

"Organizational change, evidenced through changes in job design, physical work environment, workplace social norms, or workplace culture, is likely to take longer than the four-week study timeframe to become institutionalized. However, this element was still important to include in the current study, not only to determine feasibility of delivery, but also because organizational change is necessary for sustained adoption of workplace behaviour change and control of work-related risk factors."

"Comcare has now developed and disseminated their own sedentary work practice toolkit"

Statement in paper below that qual data to determine intervention feasibility and acceptability was collected and presented in this paper...

*Multi-component intervention - targeting individual-, environmental- and organisational-level change elements*

*Management approval obtained for employee recruitment, environmental changes in the office and for study contacts to occur during worktime.*

*Recruitment email sent by management*

*Representatives were involved in brainstorming strategies, so it was organisation-specific, participants were involved in amending these strategies.*

*Participants also had personalised plans re. goal-setting etc.*

*No theory used/documented.*

*No cost-effectiveness reported - although cost of sit-stand desks included as US$400.*

**Stephens 2014: (linked to paper above)**

From discussion:

Stand up and Sit less messages seemed to be demonstrated but Move more showed minimal uptake

"Specifically, effects by time since starting work were not observed (suggesting fatigue may not be a primary driver of the changes), whereas effects of time of day were observed (suggesting that issues around how workers structure their day and their breaks may be important). Here, morning was a particularly important period of change, with the least change occurring between 12:00 and 13:00 (a common lunch period in the office environment). The correlates of these changes, including the influence of workplace, social norms and peer support, should be investigated in future research."

"The findings suggest that interventions that address both sitting bout duration and the number of sitting bouts (i.e., fewer and shorter bouts) can be effective in reducing total workplace sitting time. Furthermore, focusing on time of day rather than time since starting work may be more beneficial for adopting change across the workday."

Statement in discussion:

"qualitative data were collected to determine feasibility and acceptability of the intervention as well as the participants most favoured intervention component (reported in the main outcomes paper). However, data were not collected to qualitatively describe the context of the change to extricate the effects of the environmental strategies (i.e., the sit–stand workstations) from the organisational level support and individual behavioural change strategies. Such information may have helped to explain the wide individual variability observed."

**Healy 2016 RCT:**

From discussion:

- control group also improved in several activity outcomes - may be random findings, observer effects, and response to the feedback provided, or these may reflect general trends within the workplace. Possible interaction between worksites - ?contamination, also media attention on sitting and health risks

- "Critically the intervention elements, including tailoring, flexibility, and a participatory approach, were designed with consideration for scale-up and wider dissemination. The challenge now is to understand the uptake, implementation, and effectiveness when adapted for this next phase."

Worksites identified by a DHS appointed research liaison person

Within each worksite, a team (distinct working group within the site that had a dedicated line manager and regular group meetings and interactions was identified. Then consent was gained from the divisional manager of each team for their employees to participate, for the environmental component to be incorporated into the office, and for health coaching elements to be conducted during work time.

*Intervention development drew on social cognitive theory and an ecological model of SB*

*Participatory approach used*

*Tailoring to organisation and individual worksite's needs.*

*Description of intervention implementation provided:*

*- all worksites completed initial consultation and all team champions complied with tailored email protocol*

*- all intervention participants received their face-to-face coaching session and the associated email and at least 1 telephone call with 57% participants receiving all 4 calls.*

*- median duration of face-to-face session was 35 mins, and 8 mins for telephone calls*

*No cost-effectiveness data*

**Dunstan 2013 Protocol: (linked to paper above)**

From discussion:

"identification of the moderators of the intervention effect on workplace sitting will lead to improved understanding of which workers may be most suited to this type of intervention. These analyses may help inform targeted delivery of the intervention to specific sub-groups of workers and the appropriate adaptation of the intervention for other subgroups for which it was less successful."

"Managers will be asked to provide written informed consent and express unreserved commitment to having their employees participate in the study."

Management will send emails - "The purpose of the management emails is to foster a sense of management support for the key intervention messages."

**Neuhaus 2014 Intervention Development: (linked to papers above)**

Feedback from pilot study (not reported elsewhere as far as I can find) that informed intervention development:

Workstation group - "Overall, all five employees were satisfied with the workstations. While suggestions were made for the improvement of the workstation design, everyone appreciated the option to sit or stand while working at their computer – for example, one employee stated: “It was nice to have the option to sit or stand. It took a lot of pressure off my lower back which usually tends to get sore after prolonged periods of sitting”. On a 5-point scale (1=’did not like it at all’ to 5=’found it great’), participants rated the workstations from 3 to 5 with an average of 3.9 points. None of the participants perceived any disturbance (visual or auditory) for colleagues working in their immediate environment. Four participants expressed interest in keeping their workstation."

Peer group - "Peer group feedback Six peer group participants did not feel disturbed in any way by others using the workstations. One participant however experienced distraction through the increased noise level and the fact that the ‘workstation user’ was able to look over the partition while standing up - “We have staff come and see us about confidential/ personal information at our desks. It feels like someone is constantly staring at you”. Based on the feedback from this participant, a discussion about the purchase of cubicle dividers was taken into the protocol for the management consultation (details below) at the outset of the Stand Up Australia Intervention."

From discussion:

"Detailed reporting on intervention development is vital for the advancement of effective behaviour change interventions."

Intervention development informed by an intervention development framework - a workplace health promotion framework chosen as core approach, with elements of 2 other frameworks to complement this approach. "This included the following key elements: a phased and iterative approach in the development of the intervention [38]; the use of quantitative and qualitative evaluation methods to inform the intervention content [38]; formative research with the target group [39]; and, integration of interrelated dynamics of intra-individual, social, organisational, political, and economic factors within the workplace context [34]."

Development involved 3 stages: "1) Conceptualisation (literature review and theoretical grounding); 2) Formative research (with the target audience); and, 3) Pilot testing of the efficacy, acceptability and feasibility of the integrated multiple components relative to a control group." (Pilot testing = 3 arm trial by Neuhaus 2014 below)

Theory used:

Conceptualisation "based on social cognitive theory, which emphasises key constructs of self-efficacy, outcome expectancies (physical, social and self-evaluative) and socio-structural factors (facilitators and impediments)". Also links to socio-ecological models to emphasise the importance of targeting multiple levels of behaviour change. "In accordance with these models and frameworks, this approach included strategies designed to address organisational structures and the office environment, as well as individuals. By targeting these multiple levels, the aim was to not only raise awareness of sitting behaviours in the workplace, but also to facilitate habitual change via addressing the environment and the workplace culture."

Participatory approach used:

"this participative approach was implemented through its iterative design including formative research, brainstorming sessions and qualitative feedback interviews. This involved all levels of staff including occupational health and safety (OHS) personnel, workplace safety advisors, and corporate ergonomists (depending on the size of the targeted workplace, this includes senior- and middle managers, as well as team leaders/team champions)."

Management were initially contacted to get their support, then a representatives consultation workshop held, and brainstorming sessions for staff. During reps workshop - team champions were selected to identify behaviour change opportunities in their workplace and were involved in delivering one element of the organisational intervention components (management emails).

Clear mapping of intervention and how elements are linked to key messages is provided (table 2 of paper)

Formative research:

Organisational level - included consultation with management to gain "buy-in" plus identification of organisational processes and structures important to study implementation.

Environmental level - preliminary study conducted testing efficacy, acceptability and feasibility of sit-stand workstations (Alkhajah 2012 above), addressing some of the limitations in this study, a further study was conducted to test acceptability of sit-stand workstations in open plan offices (info in qual section of this spreadsheet).

Individual level - feasibility of face-to-face coaching sessions was tested by 2 women - overall received well.

Pilot testing: Healy 2013 (above) to test the combined implementation of all 3 intervention components (intervention refinement based on the feedback from this study is described e.g. moving from standard email from manager to tailored email, managers to initiate standing, detailed ergonomic intro to sit-stand desks included, assisting participants with stop-watches/computer software to remind them to stand) and Neuhaus 2014 (below)

**Healy 2016 Description: (linked to papers above)**

"A key theme arising from the multi-method assessment of the various Stand Up Australia evaluations [1, 11, 13] was the importance of the workplace team champion in promoting the intervention messages and strategies, and creating a supportive organisational culture for change. This finding, consistent with workplace health promotion frameworks [29], strongly informed the ‘train the champion’ approach used to adapt the Stand Up Australia intervention for wide scale delivery in collaboration with government partners"

Adaptions to program delivery:

"One of the primary requirements was for the program to be able to be delivered with no/ low cost for workplaces. Therefore, one of the key adaptions was the transferral of the administration of the program delivery and evaluation from the research team to a workplace champion (identified by the workplace). To facilitate this, a comprehensive, stand-alone, free toolkit was developed to provide workplace champions with a guide and resources to facilitate organisational buy in, and support delivery and evaluation of the program in their workplace. An online web-based format for the toolkit was chosen as it allowed for: increased reach of the program; rapid communication and evidence updates; integration of multi-media platforms; and, was a cost-effective mode of delivery. The toolkit follows a step-by-step process and uses a ‘train the champion’ approach."

Adaptions to program content:

"The materials and protocols from Stand Up Australia, alongside other evidence based research and health promotion materials and models, were used to inform the toolkit materials. Materials were adapted to follow five steps, consistent with existing guides [30]: Step 1: Getting support from management (i.e. presenting the business case and formalising commitment); Step 2: Needs assessment (i.e. assessment of workplace environment and staff health, behaviour, knowledge and attitudes prior to program implementation); Step 3: Preparing for the program (i.e. establishing a workplace wellbeing committee and running an all-of-staff information and consultation workshop to brainstorm and decide on three top stand up, sit less, move more strategies to implement in their workplace); Step 4: Putting it into practice (i.e. setting an action plan and launching the program); and, Step 5: Evaluation (assessment of program implementation; repeat of the staff survey and workplace audit).

Tools to support each of these steps (e.g. business case templates, sample policies, surveys, checklists, action plans) were developed. Other key adaptions from the original materials included the modification of wording to be less researcher oriented, more consumer friendly and understandable at the general population level, and the increased use of graphics to convey the intervention messages and goals of the program. Videos (all <10 minutes) were created as a tool for promoting buy in, raising awareness of the program and the key messages of stand up, sit less, move more, and to promote staff discussion (facilitated by the workplace champion). The evaluation framework used in the precursor trial was shortened in length and scope to be feasible for implementation without researcher input, resulting in the creation of an online, self-completion questionnaire. In addition, a component addressing the business case for reducing sitting in the workplace (not required previously in the research context), was developed. This component, which was based on the business case fact sheet developed by Comcare in 2012 [31], provides background evidence about the impacts of too much sitting, the return on investment for workplaces for supporting a healthy workplace, and suggests cost effective solutions to reduce the risks associated with too much sitting."

From discussion:

"Key elements for translation included: development of a research-government partnership leading to funding for, and collaborative adaption of, the original program for wide scale uptake; and, the integration of program buy in, delivery and evaluation systems within the online toolkit."

**Jancey 2016:**

"More research is needed to examine the impact of the office design, so that more evidence can be gathered to strengthen the argument for policy that supports the design of ‘buildings for health’. For example, although recognised as the preferred transport choice for health, stair quality, experience and use are not promoted through the National Codes of Construction, and the Property Council of Australia Guide to Office Building Quality is concerned with lift use, safety and universal access. There is no mention of encouraging stair use, which encourages movement and leads to better health outcomes for office workers in the longer term."

*Good description of environment provided*

*Single-level intervention - environmental change*

*No theory used/documented.*

*No co-production used.*

*No reported tailoring.*

*No cost-effectiveness reported.*

*But it was a natural experiment i.e. a change that was happening anyway*

**Leavy 2016 Qual: (linked to study below)**

Focus group data - 4 themes:

Enhanced general wellbeing:

- Felt more energised, less tired, more refreshed mentally, experienced a marked absence of back pain "I'm feeling so much more flexible…it’s just really good to stretch, your body needs that stretch. I feel more energetic, less pains in my back, my neck, more flexible, more awake while working."

Workability/practicality:

- Positive impacts included less email exchanges with colleagues and more face-to-face interactions "It was easier to move around, to go to talk to someone, to interact."

- Decision to stand did not disrupt their ability to concentrate on different work tasks "I was surprised I could think on my feet and that I could do tasks that required concentration either sitting or standing."

- Some were uncomfortable standing in an open plan office "Movement could be distracting. Swaying when standing, thought if I did share my office with anyone it would be strange."

Disadvantages of retro-fit workstation:

- Problems with design of workstation, awkward to use, uncomfortable

Triggers to stand:

- Time-based prompts (e.g. downloadable apps) "Used a timer, stood for ten minutes of every hour."

- Task-based factors (e.g. morning coffee or after lunch) "I always want to nap at two o'clock so I stood up and that would really push me through."

- More complex tasks or those requiring lots of paperwork was not conducive to standing

- Phone calls, emails could be undertaken with ease whilst standing

- Leaving workstation in standing position at end of day acted as a trigger the following day "...left it up and the end of day, so would come in the next day and stand. Would stand for a large proportion of the morning as would come in fresh."

- Colleagues in the office would encourage each other to stand "Others in my office with the same stand-up desk would encourage you to stand."

3 participants asked for desk to be removed due to poor design impacting on work efficiency, acute neck pain, requirement for desktop which was hindered by sit-stand desk.

Supervisor interviews - 5 themes:

Enhanced general health and wellbeing:

- Increase general health and wellbeing of employees, particularly focusing on back health care, opening up conversations about posture and occupational considerations for good health "Health benefits, occupational health, posture, back issues, the evidence supports it to improve employee overall health."

Engagement with work:

- Increased level of engagement observed "...employee outputs plus, plus, positivity, mental difference in staff member, they appeared more alert, sharper, outputs increased."

- Negative impact on employee and their workability "...was easily distracted, productivity decreased and mostly sat at desk."

Opportunity and flexibility to move more in the workplace:

- Opportunity to promote more movement at work and that the desks worked with other public health messages aimed at promoting PA at work e.g. take the stairs not the lift

- Some supervisors already had strategies to break up prolonged sitting e.g. walking emails, so sit-stand desks complimented these "We use walking emails so this reinforced the message of move more, we want to get people in our organisation to stand more so this was key."

Employee morale:

- Supervisors discussed the notion of staff as a ‘valuable resource’, and therefore the need to provide a supportive working environment. This included strategies to ensure staff felt valued and respected and supporting the creation of an organisational policy that enables all staff to access a standing station. "If you value and respect your staff they will be productive. They spend more time at work than home so [we] need to be investing in employee health and well-being and reward what they do in the workplace by having the option to stand."

Injury prevention and management:

- These were felt to be important implications for occupational health and important considerations for the organisation

- Supervisors felt that a comprehensive educational package and/or instructional video be made available and ergonomic assessment pre- and post-workstation installation

- Other comments highlighted office-space design and cost-benefit analysis prior to installation of the workstation as important considerations for an organisation "The benefits balance out any costs, and I can only see benefits, this is a best buy for Curtin."

- Whilst cost may be prohibitive for some workplaces, it was felt that "Cost is not an issue, this is the next workplace challenge and we need to tackle it."

From discussion:

"The supervisors’ suggested practices to support increased movement such as a ‘work place champion’ or ‘role model’ could be used to promote the standing stations, together with other opportunities to be break up sitting time in the workplace, such as walking emails and standing meetings. The supervisors’ believed the champions or role models could act as change agents, advocating for the allocation of resources and influence organisational policy to target sedentary behaviour in the work place."

"Furthermore, the supervisors recognised that despite an initial cost outlay, the installation of sit-stand workstations offer an opportunity for organisations to promote an ethos that is flexible, open to change, and supportive of their staff."

"Finally, supervisors highlighted the need for a business case for the purchase, installation and supporting strategies (e.g. periodic ergonomic assessments). This would ensure equitable access to the adjustable workstation by all employees and send a message that the health of employees is valued."

Permission to install the sit-stand desks was obtained from employee's supervisor

Multi-level intervention - environmental change, plus brief educational element

*No theory used/documented.*

*No co-production used.*

*No reported tailoring.*

*No cost-effectiveness reported - although supervisors did note issues with cost effectiveness.*

**Mackenzie 2015:**

n=19 completed post-intervention questionnaire

Intervention generally felt to be acceptable and feasible "the suggestions made for [the intervention] fitted with what would be possible within a work environment like [this department]."

Standing/walking meetings got a mixed response "standing/walking meetings were less appropriate as most of the work I do involves leafing through results or looking at computer screens which works better with a desk and chair"; "[this department] is quite a flexible work environment and so things like walking meetings would be acceptable."

Felt that intervention made positive impacts on workplace sitting time due to awareness raising, reminders to sit less, one regular staff meeting converted into a standing meeting.

Most helpful elements of intervention - resulted in people talking about the issues of prolonged sitting, emails from management provided supported culture to changing behaviour "emails from the Dean showed support and reminded us that we should not feel guilty taking a break."

Standing/walking meetings, posters and reminder software also felt to be helpful by some.

Unhelpful elements felt by some included reminder software, Twitter, posters.

Barriers to the intervention included: desk-based nature of work, workload/time, workplace environment, views of peers "Sometimes it looks like you're not working if you're not at your desk."

Other benefits included improved productivity/concentration, reduced stress, increased awareness of associated health implications, improved workplace culture/changing social norms, improved physical health.

Suggested improvements - ergonomic adaptations particularly standing/treadmill desks "I guess for there to be a major improvement in sitting less there needs to be some workplace changes to really make sitting less possible."

Other suggestions included: integrate with other initiatives, more "leading by example", effective use of workplace champions, financial investment, more scheduled activities, demonstrate that elements are evidence-based.

From discussion:

"Due to the need to develop low-cost interventions to support uptake amongst a variety of organisations, it was not considered appropriate to include such elements, which instead resulted in the development of a simple and pragmatic intervention."

"The main strength of this pilot was the adoption of a systematic and evidence-based intervention development process, incorporating conceptualisation and formative

research. The fact that the intervention was grounded within a theoretical model (SEM) allowed the multiple levels of influence to be targeted, thereby ensuring that: individuals' autonomy and knowledge were increased; social networks were developed; and organisational support was obtained."

"...the use of a participatory approach has been demonstrated as an effective mechanism to reduce workplace sitting, which ensured a match between the needs of the staff and the suggested strategies. The participatory approach to intervention development, ensured that the intervention was more likely to be acceptable to (and feasible for) staff. This approach allowed the development of a pragmatic intervention for use in a “real-world” setting."

*Multi-level intervention - although not really environmental change*

*Theory used/documented - SEM.*

*Co-production used.*

*Intervention tailored to the site as a result of co-production, no individual-level tailoring occurred.*

*No cost-effectiveness reported - although intervention designed was "low-cost".*

**Neuhaus 2014 RCT: (linked to Neuhaus 2014 above)**

From discussion:

"These results suggest that it is feasible to implement a multi-component intervention such as was used in Stand Up UQ with high fidelity, no perceived decrease in productivity, and few adverse outcomes. However, such study components are also resource intensive, including the installed workstations (currently retailing for approximately US$499, plus installation cost), and delivery of other intervention elements. Although the findings indicate that individual and organizational supports are important for reducing workplace sitting time, it is not possible to identify if any particular strategies were more important than others. As the individual-level intervention components are the most cost-intensive, future studies could evaluate the efficacy of the multicomponent intervention in comparison to an intervention including only height-adjustable workstations and organizational strategies."

Unit managers were given details of the study rationale and procedures, and all provided consent for their unit to participate.

Multi-component intervention:

"The intervention was based on social cognitive theory, with emphasis on self-efficacy, outcome expectancies, and sociostructural factors. The operationalization of theoretical constructs into intervention strategies was guided by an intervention taxonomy, and focused on provision of normative feedback, goalsetting, self-monitoring and problem-solving. Strategies were applied at the organizational (e.g., through group-level normative feedback in comparison to the average sitting time among Australian office workers); environmental (e.g., normative cues from co-workers standing at height-adjustable desks); and individual level (e.g., through normative individual feedback at baseline in comparison to the group’s sitting time)."

*Intervention delivery described. "Intervention fidelity was maintained through the use of detailed intervention scripts and checklists, and weekly meetings with senior study investigators."*

*Although no formal cost-effectiveness carried out - discussion section mentions high cost of multicomponent intervention*

*Theory used/documented.*

*No formal co-production used - but management involved as described above and staff involved in "brainstorming session" (see organisational intervention in intervention section).*

*Individual-level tailoring reported, also addressing workplace culture mentioned suggesting some specific organisational tailoring (see organisational intervention in intervention section).*

*No cost-effectiveness reported - although supervisors did note issues with cost effectiveness.*

**Parry 2013:**

From discussion:

"Organisations 2 and 3 involved call centre and data processing work and showed the least change in sedentary time, sustained sedentary time and break rate during work hours. In these organisations, productivity and compliance measures were monitored regularly and employees had the least amount of work flexibility and control with little opportunity to vary their work tasks or even when to take coffee and meal breaks. Therefore, in order to create meaningful and sustainable changes in sedentary time, in arguably the most challenging and sedentary group of office workers, sedentary work practices needed to change. Workplace practices within the organisations that participated in the study were regimented so that varying office tasks to incorporate incidental activity, such as longer walks to the printer were difficult to implement. Feedback from the participants indicated that these interventions were not fully supported by the management/team leaders within the organisations. Even though management and participants were aware of the intervention options, changing the organisational culture in these workplaces had limited success and such change may require stronger external support such as guidelines."

"There are number of potential reasons for why there did not appear to be one intervention that was clearly superior to the others in terms of reduced sedentary time on work days and during work hours. Participants from all intervention groups took part in workplace meetings to develop workplace specific interventions as part of the participatory approach. As a result of the consulting process, there were overlapping intervention ideas so that some of the interventions strategies implemented were common across the intervention groups. Further, the active office and physical activity interventions were very similar for most participants as only a few participants used the Active Workstation and then usually only to a limited extent. Feedback from the participants indicated barriers to use of the Active Workstation included the time taken to log on and off their regular computer, an unfamiliar workstation and fear of perceived loss of productive work time."

"The success of each of the interventions may also be indicative of the participatory approach ensuring a match between the work group and the variety of strategies available to encourage occupational incidental activity and reduced occupational sedentary time."

Good clear descriptions of organisations involved in the study to provide useful context.

Interventions were developed using a participatory approach. Participants from all 3 interventions were asked to attend 2 structured meetings in their workplace to discuss and develop interventions. Made sure interventions were tailored to the specific needs of the workplace and the employee participants had ownership.

During 1st meeting - participants brainstormed options to promote their specific intervention

In between meetings - encouraged to think about specific strategies

At 2nd meeting (2-3 weeks after 1st) - participants shared their ideas and rated the potential strategies in terms of feasibility and effectiveness, then action plan developed and facilitator communicated with team leaders and management to help implementation

Throughout intervention period to communicate with and motivate participants, tailored emails were sent to each participant by a facilitator every 2-3 weeks.

*Multi-level interventions*

*No theory used/documented.*

*Participatory approach used - therefore some organisation tailoring*

*No cost-effectiveness reported.*

**Pronk 2011:**

Open ended questionnaire responses - "less low back pain and shoulder tension, posture improvement, decreased wrist and elbow pain, and increased comfort. No negative comments were received."

From discussion:

"interventions that influence various aspects of the work environment (e.g., physical, psychosocial) and focus on an integrated approach to worker health protection and promotion, such as the Take-a-Stand Project, may have benefits that exceed those gained by initiatives focused on only 1 approach."

"Our project considers the physical and psychosocial environments by redesigning workstations to include a sit-stand device for the employee." ?how this is psychosocial

Whole study was within context of ongoing multicomponent health and wellbeing programme.

"Supervisors and managers actively supported the project’s implementation"

*Single-level interventions - although was part of a larger general health and wellbeing programme*

*No theory used/documented.*

*No co-production.*

*No cost-effectiveness reported.*

**Puig-Ribera 2015:**

From discussion:

"Walk@Work represents a program with in-built flexibility that can adapt to local environmental and socio-cultural conditions"

"W@WS represents a low-cost automated programme, implemented by employees without the need to change the office environment"

Single-level intervention, but multi-component

No theory used/documented - although in the discussion states that intervention is "theoretically derived"

No co-production.

No cost-effectiveness reported - although in discussion stated that intervention was low-cost

**Urda 2016:**

From discussion:

"A possible explanation [for a lack of within group differences in sitting time in CI group] was revealed through anecdotal reports from participants who offered that they became more aware of theory workplace standing and sitting behaviours and may have inadvertently reduced workplace sitting during the control week."

"Anecdotally, some participants offered that by wearing the activPAL3 activity monitor, they became more aware of their standing and sitting behaviours at work and suggested they may have increased their sit-stand transitions per workday during the control week. Further, several participants reported that, as part of their normal daily job tasks, they needed to stand up numerous times per day to work with students. Therefore, any potential change resulting from the nontailored intervention used in this study may have been overshadowed by the number of times they were already getting out of their chairs throughout the workday."

"Management gave full support for those who participated in this study."

**Priebe 2015:**

From discussion:

"Based on theory and current empirical findings in other areas, it was hypothesized that high contextual and personal similarity would produce greater behaviour change than conditions reflecting lower contextual (e.g., Goldstein et al., 2008) and lower personal similarity (e.g., Rimal & Real, 2005). The failure to find differences between the similarity conditions might reflect that these factors are not salient in the activity or office setting (i.e., descriptive norms are effective regardless of the similarity of the reference group). However, the fact that a strong relationship between descriptive norms and other types of activity behaviour has only emerged in past research when norms were framed around specific reference groups (Priene & Spin, 2011) makes this explanation less tenable."

"A more plausible explanation relates to the fidelity of the delivered manipulation in this field experiment. Manipulation checks revealed that participants who recalled receiving the messages varied in their recall of the reference group similarity information. Just over half of the participants were able to accurately recall the contextual (office location; 56%) or the personal similarity information (reason for being active; 64%) in their condition. Given this reported inaccuracy in recall, the present results do not provide a strong test of the proposed hypotheses regarding differences due to groups that varied in similarity. It is possible results would be different if participants processed the messages as intended."

*Descriptive norms are grounded in theory and empirical evidence (described in intro)*

*Single-level intervention*

*No co-production.*

*No cost-effectiveness reported - although in discussion stated that intervention was low-cost*

**Gordon 2013:**

"Post-test assessment of productivity and acceptability of the intervention found that 92% of the intervention group participants found the overall intervention to be acceptable. The majority of participants in the intervention group felt that sitting less increased or highly increased the focus (80%), quality (70%), and productivity (70%) of their work as compared to 50% of the control group, who felt that sitting less had no impact on their work performance"

From discussion:

"In light of the insignificant change in sitting time it appears likely the intervention intensity may have been insufficient given the heterogeneous workplaces involved and the extended duration of the study. One of the central components of social cognitive theory is the idea of reciprocal determinism, meaning factors from a variety of spheres play a part in instigating and maintaining change. In this case researchers hypothesize that an intervention which targets not only the individual, as was done here, but the social and physical environments may elicit a greater and more significant change in long term sitting behaviours at work. Qualitative studies in employee perceptions found that employees who know that their superiors support and or encourage taking standing and or walking breaks may be more likely to practice these behaviours (65). Similarly, given a physical environment where standing is possible without incurring injury from improper mechanics, such as hunching over a desk to see a screen while standing may further encourage standing and stepping behaviours"

"We therefore cannot definitively state whether a sedentary behaviour intervention has any positive impact on perceptions of workplace productivity. However, all participants reported either “no impact on” or a variation of “increases” productivity indicating that instigating this program had no negative consequences in perceptions of productivity."

"In qualitative interviews following the completion of the program participants expressed high acceptability of the intervention and many requested further information to aid in “bottom up” approaches to policy and environmental changes in their worksite, citing a desire to have physical access to alternatives to sitting at a desk all day. Though assessment of worksite policy was beyond the scope of this study, it is encouraging that participants would take the message of this program to heart in lobbying for change individually, which may further facilitate greater change in the future. Though individualized programs have been effective in eliciting initial change, additionally changing some aspect of the environment, especially one so focused on efficiency as opposed to health, may be equally if not more important for lasting behaviour change in the workplace."

*Used social cognitive theory to guide intervention material development*

*Also used the RE-AIM framework (Reach, Efficacy/Effectiveness, Adoption, Implementation, Maintenance) to assess ability of prospective setting to sustain the program once study is complete.*

*?Single-level intervention - behaviour change but also mention of a walking workstation and unclear if this was used as part of the intervention in which case would class as environmental element*

*No co-production.*

*No cost-effectiveness reported*

**Richards 2015:**

The majority of staff felt strongly that it was expected of them and felt social pressure to sit for prolonged periods. At follow-up, the median score for these items had decreased. Staff disagreed that their important others at work think that they should sit for prolonged periods and this remained unchanged at follow-up.

At baseline, staff reported some confidence that they could reduce the amount of time they spend sitting during working hours. Staff did not perceive the task of reducing their sitting time at work to be easy. At follow up, staff were unsure whether the time they spent sitting at work was beyond their control and there was an increase in agreement that whether they reduce the amount of time spent sitting or not was up to them.

At baseline, staff described six potential barriers that might prevent them from reducing their sitting time during working hours (Table 6). The majority of staff anticipated the main barrier to be their need to use the computer or be at their desks for prolonged periods, due to the nature of their work activities. One member of staff commented, “All my work is on the computer, so I need to sit down’ and another, ‘It is difficult to stand and write”. Many staff perceived that it is the norm to sit for prolonged periods of time and sit during meetings and presentations and as such, felt that social pressure to sit might be a barrier to reducing their sitting time. One member of staff commented, “If I am standing and others are sitting, it feels like a strange power imbalance”. Others perceived that their high workload meant that they were too busy to engage in strategies to reduce their sitting time and one member of staff suggested that they forget to engage in strategies, possibly due to a high workload. Some staff were concerned that reducing their sitting time would decrease their productivity and some felt that they lacked the equipment to facilitate a reduction in sitting time, such as sit-stand workstations.

At baseline, staff described five potential facilitators that might help them to reduce their sitting time during working hours (Table 7). The majority of staff suggested that having suitable equipment, such as a standing desk, would help to reduce their sitting time by allowing them to carry out their computer- and desk-based activities without having to sit for prolonged periods of time. Other equipment suggestions included fitness-equipment and cordless telephones. Three potential facilitators involved changing the workplace environment. Many members of staff suggested that having more opportunities to reduce sitting time at work would help, for example a lunch time walking group or standing/walking meetings. Many also perceived that changing the social norm in the workplace so that it is more acceptable to reduce sitting time would help them to reduce their sitting time. One member of staff commented that they would like to see “other people taking a break for lunch”. Other potential facilitators included the use of reminders to prompt staff to reduce their sitting time and the encouragement of their manager to support them in their efforts to reduce their sitting time, to reduce feelings of guilt when being away from their desks.

At follow-up, staff described six barriers that they perceived prevented them from reducing their sitting time during working hours (Table 8). The majority of staff perceived that the main barrier to reducing their sitting time was their need to use the computer or be at their desks for prolonged periods, due to the nature of their work activities. Many members of staff felt social pressure to sit for prolonged periods during work activities, such as in meetings, and one member of staff commented, “I was worried about causing distractions or making my colleagues feel uncomfortable”. Others perceived that forgetting to take a regular break to reduce their sitting time was a barrier, possibly due to a high workload. One member of staff commented, “I forget to take a break when working intensely”. Another barrier to reducing sitting time was concerns about being less productive, with one member of staff commenting, “I found it distracting to have to think about it whilst trying to concentrate on my work”. Other barriers included the high workload of staff, with work being their “priority”, and a lack of equipment to facilitate a reduction in sitting time, such as standing desks or cordless telephones.

At follow-up, staff described five potential facilitators that had helped them to reduce their sitting time during working hours (Table 9). Many members of staff reported that they found the daily email reminders useful as they prompted staff to reduce their sitting time. Others perceived that due to many of their colleagues using the same strategies as them to reduce sitting time, there was a change in the social norm of sitting in the office and this helped to reduce their sitting time. Other facilitators included social support and encouragement from their colleagues to reduce sitting time and additional opportunities to reduce sitting time such as joining the lunchtime walking group. One member of staff also reported that they had purchased a wireless Dictaphone so that he could walk while using it, instead of sitting down. A further 12 participants reported that carrying out the OYFB recommended strategies helped them to reduce their sitting time.

Staff described experiencing several benefits from reducing their sitting time during working hours (Table 10). Many members of staff reported feeling better in some way, for example one member of staff reported feeling “refreshed”, others felt “less stiff” and most reported simply feeling “better”. Others reported being more active and some felt more productive. One member of staff commented that reducing their sitting time, “cleared my head, I was more able to concentrate”. In contrast, some members of staff reported that they had not noticed or were unsure if they had experienced any benefits.

From discussion:

"The results of the current study also suggest that organisations should consider which strategies are suitable for its environment. For example, in the current study, it was not feasible to encourage staff to eat lunch away from their desks as the communal kitchen is very small and not able to accommodate all staff at the same time. Similarly, it was not feasible for all staff to stand at the back of the room during a presentation."

"The social norm of sitting for prolonged periods of time at work was also reported as a barrier to reducing sitting time. Feelings that there was an expectation and social pressure to sit for prolonged periods remained at follow up however there was a decrease in these perceptions. Many members of staff in the same department took part in the intervention, which may have normalised the strategies that they were using to reduce their sitting time and legitimised the target behaviour as they were all attempting to reduce their sitting time together as a group."

"The intervention also included encouragement and support from the departmental director, which may have contributed to the reduction in the expectation to be sitting for prolonged periods."

"The current study showed that staff already had a high awareness of the harmful effects of sitting and intentions to reduce their sitting time, therefore it is likely that academics are prioritising workload over their health. In sum, academics appear to have the psychological capability (knowledge) to reduce their sitting time, however they have limited physical opportunity (due to high workload) and social opportunity (due to perceived social norms of sitting) to do so. Future interventions are needed at the organisational and policy level in order to provide academic staff with increased opportunity to reduce their sitting time."

Prior to the presentation, the director of the department provided verbal approval of the intervention and motivated and encouraged staff to reduce their sitting time at work.

Intervention development guided by Behaviour Change Wheel:

A behavioural analysis was conducted to develop an intervention to reduce the sitting time of academic staff at work. Capability (psychological), Opportunity (physical and social) and Motivation (reflective and automatic) were selected as sources of behaviour to change in order to achieve a reduction of sitting time. The Theoretical Domains Framework (TDF) (Michie et al., 2005) was used to further outline which elements of the COM-B model components would be specifically targeted.

*Single-level intervention - behaviour change but with multiple components*

*No co-production.*

*No cost-effectiveness reported - although in conclusion does state intervention is low-cost*
